# Antifreezing and Temperature-Responsive Ionic Hydrogels with Applications in Encryption and Sensor Technologies

**DOI:** 10.1021/acsami.5c08600

**Published:** 2025-07-14

**Authors:** Xia Qiu, Xiaolong He, Kubra Kalayci, Paul Morandi, Petra Rudolf, Rudy Folkersma, Vincent S. D. Voet, Katja Loos

**Affiliations:** † Macromolecular Chemistry and New Polymeric Materials, Zernike Institute for Advanced Materials, 3647 University of Groningen, Nijenborgh 3, 9747 AG Groningen, The Netherlands; ‡ Surfaces and Thin Films, Zernike Institute for Advanced Materials, University of Groningen, Nijenborgh 3, 9747 AG Groningen, The Netherlands; § Circular Plastics, Academy Technology & Innovation, 84808 NHL Stenden University of Applied Sciences, Van Schaikweg 94, 7811 KL Emmen, The Netherlands

**Keywords:** thermoresponsiveness, antifreezing, biobased hydrogel, wearable sensor, encryption

## Abstract

The use of thermoresponsive hydrogels for applications such as sensors, thermal gates, smart windows, actuators, and molecular devices has increased in popularity in the past decade. However, existing thermoresponsive biobased hydrogel sensors face challenges in rapidly responding to ambient temperature changes and retaining flexibility at subzero temperatures. To overcome these limitations, a novel hydrogel composed of dextrin, glycerol, and the ionic liquid monomer tetrabutylphosphonium styrenesulfonate (PSS) was developed and utilized as a smart sensor material for the first time. The thermoresponsive characteristics of PSS endow the hydrogel with remarkable thermoresponsiveness, which is a lower critical solution temperature (LCST)-type phase transition. In addition, the hydrogel can be used as a thermally responsive material over a broad temperature range of 20–60 °C. We used glycerol and glycidyl methacrylate dextrin (Dex-GMA) monomers with a multihydrogen bond structure to construct a Dex-GMA-PSS conductive hydrogel with antifreeze properties even at −20 °C. Hence, the hydrogels formulated in this study exhibit promising potential for several applications, including flexible wearable devices, skin-like sensors, advanced anticounterfeiting, and encryption technologies across a broad temperature range.

## Graphical abstract

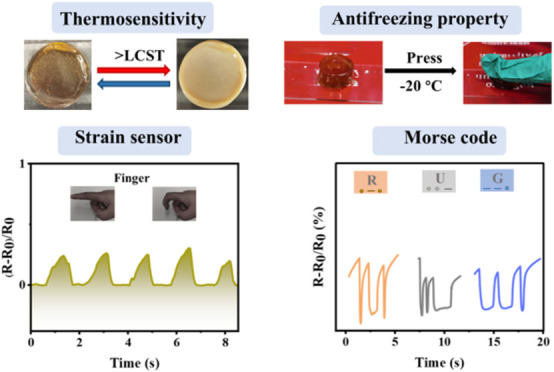

## Introduction

Hydrogels are (bio)­materials that can be naturally adaptable, conductive, and biocompatible; nonetheless, their high water content causes swelling, freezing, and dehydration, restricting their application in low-temperature environments. These challenges have spurred efforts to create antifreezing hydrogels (ATHGs), particularly for applications such as flexible sensors.^1–3^ In nature, organisms, such as peeper frogs, produce antifreeze substances, such as alcohol, to survive in cold climates.^4^ Inspired by this natural strategy, the addition of antifreeze biobased chemicals such as glycerol to hydrogels can improve performance at subzero temperatures.^5^

Another critical feature of hydrogel sensors is their responsiveness to environmental stimuli. To meet these criteria, stimuli-responsive polymers are valuable because they can undergo phase transitions in response to environmental changes, such as changes in temperature^6^ and pH.^7^ Thermoresponsive polymers have attracted a great deal of interest due to their thermal sensitivity and potential applications as “smart” materials in sensors,^8^ biomedical materials,^9^ and separation membranes.^10^ However, traditional polymers with lower critical solution temperatures (LCSTs), such as poly­(*N*-vinyl caprolactam) (PVCL),^11–13^ poly­(*N*-isopropylacrylamide) (PNIPAM),^14–17^ and poly­(vinyl methyl ether) (PVME),^18–20^ face limitations due to their fixed phase transition temperatures and generally weak or neutral charges in water, reducing their versatility and responsiveness in various adaptive applications. Thermoresponsive poly­(ionic liquids) (TRPILs), fully charged polymers composed of cations and anions, have recently gained significant attention for their versatile applications.^21–23^ By adjusting the balance of hydrophilicity and hydrophobicity between their ionic components, the LCST of these PILs can be finely tuned, allowing precise control over their temperature responsiveness.^24–27^ As a milestone, in 2012, Ohno’s group created poly­(tetrabutylphosphonium styrenesulfonate) P­(PSS), the first synthetic TRPIL.^28^ The system displays remarkable temperature responsiveness and the unique ability to extensively modify the cloud point (Tcp) via salt inclusion. The introduction of TRPILs into the hydrogel network can endow it with both temperature responsiveness and enhanced conductivity.^29^ When heated, TRPIL hydrogels exhibit a reversible transparent–opaque transition. Unlike traditional phase change materials (PCMs), such as paraffin wax, which rely on phase transitions to regulate temperature,^30^ TRPIL hydrogels gain thermal stability and mechanical strength primarily from their unique ionic interactions and cross-linked structures. This intrinsic design provides enhanced durability and responsiveness, distinguishing TRPIL hydrogels as advanced alternatives to conventional PCMs for thermal management applications. While they have been explored in energy-saving devices and smart windows,^31–34^ the potential of TRPIL hydrogels for applications in wearable sensors and multilevel encryption remains largely untapped, especially for operations across wide temperature ranges, including subzero environments. Recent studies have integrated thermal responsiveness with antifreezing properties. Liu et al. prepared an ionic hydrogel using a water/[EMIm]Cl solvent system, resulting in a flexible, adhesive, antifreezing, and temperature sensor, though it lacked optical responsiveness for encryption applications.^35^ Lopez-Larrea et al. developed a PNIPAM/PEDOT/PSS hydrogel with LCST-based drug release but limited conductivity and low-temperature stability.^36^ In contrast, TRPIL-based hydrogels offer a tunable thermal response and intrinsic conductivity, making them promising for multifunctional applications under extreme conditions, including wearable electronics and information encryption.

Polysaccharides, as renewable biopolymers, have attracted considerable research interest because of their versatile molecular structures, which support various modifications^37–40^ for creating eco-friendly, sustainable materials. These properties enable applications in self-powered touch-sensing devices,^41^ as stabilizers in food, and in environmental fields such as water purification and biodegradable packaging.^42^ Among these, polysaccharide-based hydrogels, particularly those derived from thermoresponsive polymers, offer added benefits due to their biocompatibility, biodegradability, and responsiveness to external stimuli.^43^ Dextrin (Dex), a versatile polysaccharide, is often modified with glycidyl methacrylate (GMA) to enhance its mechanical properties, water stability, and reactivity, enabling further functionalization for advanced biomedical applications.^44^

In this study, we synthesized hydrogels by polymerizing and cross-linking a thermally sensitive ionic liquid (PSS) with glycidyl methacrylate dextrin (Dex-GMA) via free-radical polymerization. This approach was designed to enhance the mechanical properties of the hydrogels while preserving their temperature responsiveness and conductivity.

The hydrogels were prepared in a glycerol/water solvent system, which leveraged strong hydrogen bonding to prevent freezing and reduce evaporation. This formulation improved the durability and broadened the potential applications of the hydrogels.

We systematically evaluated the transparency, thermal responsiveness, swelling behavior, compressive strength, and conductivity of the hydrogels across various Dex-GMA-PSS ratios. The thermoresponsive properties of the poly­(ionic liquid) (PIL) hydrogels were highlighted with particular emphasis on their innovative applications in wearable sensors and information encryption.

The resulting Dex-GMA-PSS-glycerol (DGPSSG) hydrogel demonstrated flexibility, freezing tolerance, and the ability to detect temperature and pressure. Characterization through Fourier transform infrared spectroscopy (FTIR), DSC, TGA, and rheological tests confirmed its performance, marking a significant advancement in flexible device technology.

## Experimental Section

### Materials

Dextrin (Dex) D4657, glycidyl methacrylate (GMA, 99%), ammonium peroxydisulfate (APS, 98%), *N*,*N*,*N*′,*N*′-tetramethylethylenediamine (TEMED, >98%), and 4-dimethylaminopyridine (DMAP, ≥99%) were purchased from TCI Europe. Tetrabutylphosphonium bromide ([P_4444_]­Br, 98%), sodium p-styrenesulfonate hydrate (Na­[SS], ≥90%), glycerol (≥99.5%), dimethyl sulfoxide (DMSO, ≥99.7%), dichloromethane (DCM, ≥99%) and deuterium oxide (D_2_O, 99.9%) were obtained from Sigma–Aldrich. Deionized (DI) water (18 MΩ·cm) was used throughout the experiments.

### Synthesis of Dex-GMA and Tetrabutylphosphonium p-Styrenesulfonate (PSS) Ionic Liquid Monomers

Glycidyl methacrylate dextrin (Dex-GMA) was synthesized via a previously described method from van Dijk-Wolthuis et al.^45^ In summary, 2.025 g of dextrin was dissolved in 20 mL of DMSO at room temperature. Once fully dissolved, 0.765 g of DMAP and 1.67 mL of GMA were added to the solution, which was maintained at 50 °C under ambient conditions and stirred continuously for 48 h. Hydrochloric acid (HCl) was subsequently added to neutralize the DMAP. The resulting mixture was dialyzed against distilled water for 1 week via a dialysis membrane with a molecular weight cutoff (MWCO) of 3.5 kDa. Finally, the product was freeze-dried for 48 h, yielding the Dex-GMA derivative as pale yellow–brown flakes.

Tetrabutylphosphonium p-styrenesulfonate (PSS) was synthesized via an ion exchange method.^21^ In this process, 10 g of tetrabutylphosphonium bromide and 6.5 g of sodium p-styrenesulfonate were dissolved in 40 mL of deionized (DI) water. The homogeneous solution was stirred for 12 h. The ionic liquid was extracted with 80 mL of dichloromethane, which was subsequently washed with 20 mL of DI water 3 times to remove NaBr in DCM. Then, the remaining water in the dichloromethane layer was absorbed using anhydrous magnesium sulfate before a rotary evaporator was used to remove the majority of the dichloromethane layer, and any residual liquid was removed under vacuum at room temperature for 3 h. To prevent polymerization, PSS was stored at −4 °C.

### Preparation of the Poly­(ionic liquid) Thermoresponsive Hydrogel (DGPSSG)

Initially, 0.06 g of Dex-GMA powder was dissolved in a mixture consisting of 1 mL of glycerol and 3 mL of PSS solution (10, 20, and 40% w/v), maintaining a 1:3 ratio at varying concentrations ranging from 10 to 40% (w/v) in a flask. The mixture was stirred for 10 min to achieve a homogeneous solution. Next, 0.01 g of the radical initiator APS and 24 μL of the accelerator TEMED were added to the solution, followed by sonication and free-radical polymerization, specifically in redox-initiated polymerization reactions at room temperature. The detailed parameters are provided in Table S1 (in the Supporting Information). The resulting mixture was poured into a mold, and after 1 h, the mold was removed, yielding DGPSSG hydrogel containing glycerol and DGPSS hydrogels without glycerol, each with varying ionic liquid concentrations.

### Characterization

Nuclear magnetic resonance (NMR) spectra were collected at 25 °C using a Bruker Advance 400 MHz NMR spectrometer with deuterium oxide (D_2_O) as the solvent. For proton nuclear magnetic resonance (^1^H NMR) measurements, sample solutions were prepared at a concentration of 5 mg/mL and 128 scans were recorded. Attenuated total reflectance Fourier transform infrared (ATR-FTIR) spectra were collected by using a Bruker Vertex 70 infrared spectrometer equipped with a diamond ATR probe. Each spectrum was obtained by averaging 64 scans with a resolution of 4 cm^–1^ over the spectral range of 4000–400 cm^–1^. The freezing temperatures of the DGPSS and DGPSSG hydrogels were determined via differential scanning calorimetry (DSC) using a TA Instruments Q1000 analyzer. Measurements were conducted under a nitrogen flow of 50 mL/min. The cooling cycle was performed from 20 to −60 °C at a rate of 5 °C/min under a nitrogen atmosphere. Thermogravimetric analysis (TGA) was conducted using a TA Instruments 5500 analyzer under a nitrogen atmosphere. Samples were analyzed over a temperature range of 30–700 °C at a rate of 10 °C/min. Optical images were obtained to analyze the thermoresponsiveness of the DGPSSG hydrogel. After being heated, the hydrogel was investigated using an optical microscope. The morphological properties of DGPSSG hydrogel were studied using scanning electron microscopy (SEM) on an FEI Nova NanoSEM 650 with a 15 kV accelerating voltage. Before the measurement, the hydrogels that were at 20 °C or under equilibrium in 60 °C water were quickly frozen in liquid nitrogen. The gels were then lyophilized to thoroughly dehydrate without altering the hydrogel’s inherent porous structure.

### Measurement of the Swelling Ratio of the Hydrogel

Freshly prepared hydrogel samples were weighed to determine their initial mass (0 h) before being immersed in water. The samples are then immersed in deionized water for a set time before being weighed again. The hydrogel was wiped with filter paper to remove excess water from the surface and weighed carefully at various soaking times. The swelling ratio (SR) was calculated with eq 1:

1 $$\mathrm{SR}\;\left(\%\right)=\frac{M_{i}-M_{0}}{M_{0}}\times 100$$

where *M_i_* represents the mass of the hydrogel after *i* hours of swelling, whereas *M*_0_ represents the initial mass of the hydrogel before being immersed in water.

### Water Retention Tests

Cylindrical DGPSSG hydrogels (8 mm in diameter and 8 mm in height) were incubated at room temperature for 7 days. The initial weight of the hydrogels was recorded as *W*_0_, and *W*_*t*_ represents the weight at specific intervals. The water content of the hydrogels was calculated via eq 2:

2 $$w\mathrm{ater retention}\;\left(\%\right)=\frac{W_{t}-W_{0}}{W_{0}}\times 100$$

### Rheological Test

The rheological properties of the DGPSS and DGPSSG hydrogels (25 mm in diameter and a gap of 1 mm) were studied via an Anton Paar MCR 302e Rheometer (Anton Paar, Ashland, VA) with parallel plate geometry and a diameter of 25 mm. Under each hydrogel condition, frequency sweeps were carried out from 1 to 10 Hz at 25 °C. The temperature-sweep tests for antifreezing were conducted at a frequency of 6.28 rad/s and a constant strain of 1%. The samples were initially cooled at a rate of 3 °C/min from 20 to −65 °C. The heating process for the thermoresponsive test was performed at the same rate from 20 to 70 °C. The storage modulus (*G*′) and loss modulus (*G*″) were determined. The rheological behavior of thermally reversible hydrogels was evaluated using a dynamic temperature-sweep test, with storage modulus (*G*′) and loss modulus (*G*″) measured at an angular frequency of 6.28 rad/s and a strain of 1% over 600 s. Moduli variations were analyzed following thermal cycling between 20 and 60 °C.

### The Creep-Recovery Experiment

The DGPSSG samples were subjected to an increasing stress, which was held constant for 5 min at a maximum pressure of 5 Pa. After this period, the applied stress was abruptly removed. The subsequent strain recovery, resulting from the reformation of the internal hydrogel structure, was recorded. The elastic recovery ratio of the DGPSSG samples was then quantitatively evaluated to assess their ability to recover from deformation.

### Antifreezing Experiment

To assess the antifreezing properties of the DGPSSG hydrogels, cylindrical wet samples (8 mm in diameter and height) were stored at −20 °C for 4 h. Then removed and immediately examined to assess their elasticity and performance characteristics, including conductivity. Further evaluation of the freezing resistance of the hydrogels was conducted through differential scanning calorimetry (DSC), with the temperature gradually decreasing from 20 to −60 °C at a rate of 10 °C/min. Additionally, rheological tests were performed by lowering the temperature from 20 to −65 °C at a rate of 3 °C/min to analyze their structural integrity under subzero conditions.

### Compressive Test

The mechanical properties of the hydrogels were evaluated by using a universal testing machine (Instron 68SC-1, 0.02 N load cell). For compression testing, hydrogel samples were molded into cylindrical shapes (16 mm in diameter × 6 mm in height). Tests were conducted at a speed of 2 mm/min at both 20 and −20 °C. For cyclic compression testing, a speed of 20 mm/min was used. Each group of samples was tested 3 times.

### Conductivity Characterization

The conductivity (σ) of the DGPSSG hydrogel was obtained via an electrochemical workstation. All samples were prepared as uniform cylinders 16 mm in diameter and 1 mm in height. The resistance of the hydrogel sensor was estimated via Ohm’s law (*R* = *U*/*I*), which compares the supplied constant voltage to the immediate electrical current at different temperatures (−20, 20, and 60 °C). The electrical conductivity was determined according to the following equation:

3 σ=L/(R×S)

where *L* is the length (cm), *R* represents the resistance of the hydrogel (Ω), and *S* is the cross-sectional area of the hydrogel (cm^2^).

### Fabrication and Sensing Behavior of the Wearable Sensor Based on the DGPSSG Hydrogel

The compressive sensor consisted of a cylindrical DGPSSG hydrogel (16 mm in diameter × 6 mm in height) with two copper wires attached to either end using copper tape. The sensor was tested under various strains at speeds ranging from 2 to 20 mm/min at both 20  and −20 °C. For the wearable sensor was composed of a square DGPSSG hydrogel (5 × 5 × 0.5 mm^3^), with two copper wires attached to either end via copper tape. This series-connected hydrogel was placed on various parts of the human body, including the fingers, wrists, elbows, knees, throat, corners of the mouth, and brow. Electrical signals are transmitted through the motions of these body parts to facilitate wearable sensing. The sensing performance of the hydrogel was evaluated by using an Agilent 34410A multimeter. When the hydrogel was deformed, the device recorded real-time changes in the resistance. The ratio of the resistance change was calculated via the following equation:

4 $$\frac{\Delta R}{R_{0}}\left(\%\right)=\frac{R-R_{0}}{R_{0}}\times 100$$

*R*_0_ is the resistance of the hydrogel when it is not stretched, whereas *R* is the resistance of the hydrogel that is stretched.

Furthermore, the sensitivity of the hydrogel sensors was quantified using the Gauge Factor (GF), calculated as

5 $$\mathrm{GF}=\frac{\Delta R/R_{0}}{\varepsilon }$$

where Δ*R* denotes the resistance change and ε represents the applied strain. This parameter provides a precise characterization of the hydrogel’s electromechanical transduction properties under dynamic conditions.

## Results and Discussion

### Design, Preparation, and Characterization of the DGPSSG Smart Hydrogel

Figure S1a illustrates the preparation processes for the ionic liquid PSS and Dex-GMA, which serve as essential monomers in the DGPSSG hydrogel network. The structures of Dex-GMA and PSS were confirmed by FTIR and ^1^H NMR spectroscopy.

Dex-GMA was synthesized via attaching GMA to dextrin using DMAP and an aprotic solvent (DMSO) at room temperature for 48 h. In the FTIR spectrum of Dex-GMA (Figure S2a in the Supporting Information), new characteristic bands were observed, distinguishing it from the original dextrin. These include the stretching vibrations of the ester carbonyl group (C=O) at 1722 cm^–1^ and the stretching vibration of the double bond (C=CH_2_) is observed at 1631 cm^–1^, while the bending vibration of the double bond appears at 813 cm^–1^.^45^ Additionally, the disappearance of the epoxy peaks at 910 cm^–1^ (C–O–C symmetric or asymmetric ring stretching) and 1250 cm^–1^ (C–O bond stretching) confirms the successful modification of dextrin to Dex-GMA.^45^ The ^1^H NMR spectrum (D_2_O) of Dex-GMA (Figure S2b) further supports this, showing peaks at 5.66 and 6.07 ppm for the vinyl group (CH_2_=C) and at 1.84 ppm for the methyl group of GMA. The degree of substitution (DS) of GMA on dextrin was calculated from 1H NMR spectrum, using the following equation:^45^

6 $$\mathrm{DS}=\frac{100x}{y}$$

where *x* corresponds to the average integral of the vinyl double bonds 5.66 and 6.07 ppm (Hb,c) and *y* is the integral of anomeric protons (Ha) 4.95–5.38 ppm. Based on this calculation, the DS was determined to be 43%, confirming successful derivatization of dextrin, which enables subsequent polymerization processes.

PSS was synthesized via an ion exchange method, and the FTIR spectra of [P_4444_]­Br, Na­[SS], and PSS at room temperature (Figure S3a in the Supporting Information) confirmed the successful synthesis. The peaks at 2870 and 2960 cm^–1^ are attributed to the asymmetric and symmetric C–H stretching vibrations of the alkyl group. Additionally, the asymmetric and symmetric S=O stretching vibrations at 1189 and 1120 cm^–1^ confirmed the ion exchange between [P_4444_]Br and styrenesulfonate.^46^ The ^1^H NMR spectrum of PSS (Figure S3b in the Supporting Information) shows distinct peaks corresponding to the methyl (−CH_3_) and methylene (−CH_2_) groups of [P_4444_]­Br, as well as vinyl (C=CH_2_) and aromatic (Ar–H) protons on the benzene ring. Peaks Ha, Hb, and Hc in Na­[SS] are preserved after ion exchange, further confirming the successful synthesis of PSS.^46^

The production of the DGPSSG hydrogels involved dissolving Dex-GMA in a glycerol-PSS solution, adding polymerization agents, and processing the mixture to produce hydrogels with varying ionic liquid concentrations (Figure 1a,b). FTIR analysis confirmed the successful synthesis of the DGPSSG hydrogel (Figure 1c). The spectrum displayed a characteristic C=O stretching vibration at 1722 cm^–1^, indicating the incorporation of Dex-GMA segments. Additionally, the disappearance of the bending vibration peak at 813 cm^–1^, which is associated with C=C bonds, further supports the successful formation of the polymer network.^45^ In contrast, the C=C stretching vibration peak became significantly weaker, indicating that the majority of vinyl double bonds were consumed during the radical polymerization process. The peaks at 1189 and 1120 cm^–1^ were attributed to the S=O group in PSS, confirming the presence of the ionic liquid within the hydrogel matrix. Additionally, the OH stretching vibration peak shifts from 3380 cm^–1^ (Dex–GMA) to 3359 cm^–1^ in the DGPSSG hydrogel, indicating a more constrained hydrogen bonding environment. This red-shift may result from hydrogen bonds between hydroxyl groups and polar groups (SO_3_^–^) in the polymer network.^47,48^ These spectral features provide clear evidence of the successful synthesis of the DGPSSG hydrogel.

**Figure 1:**
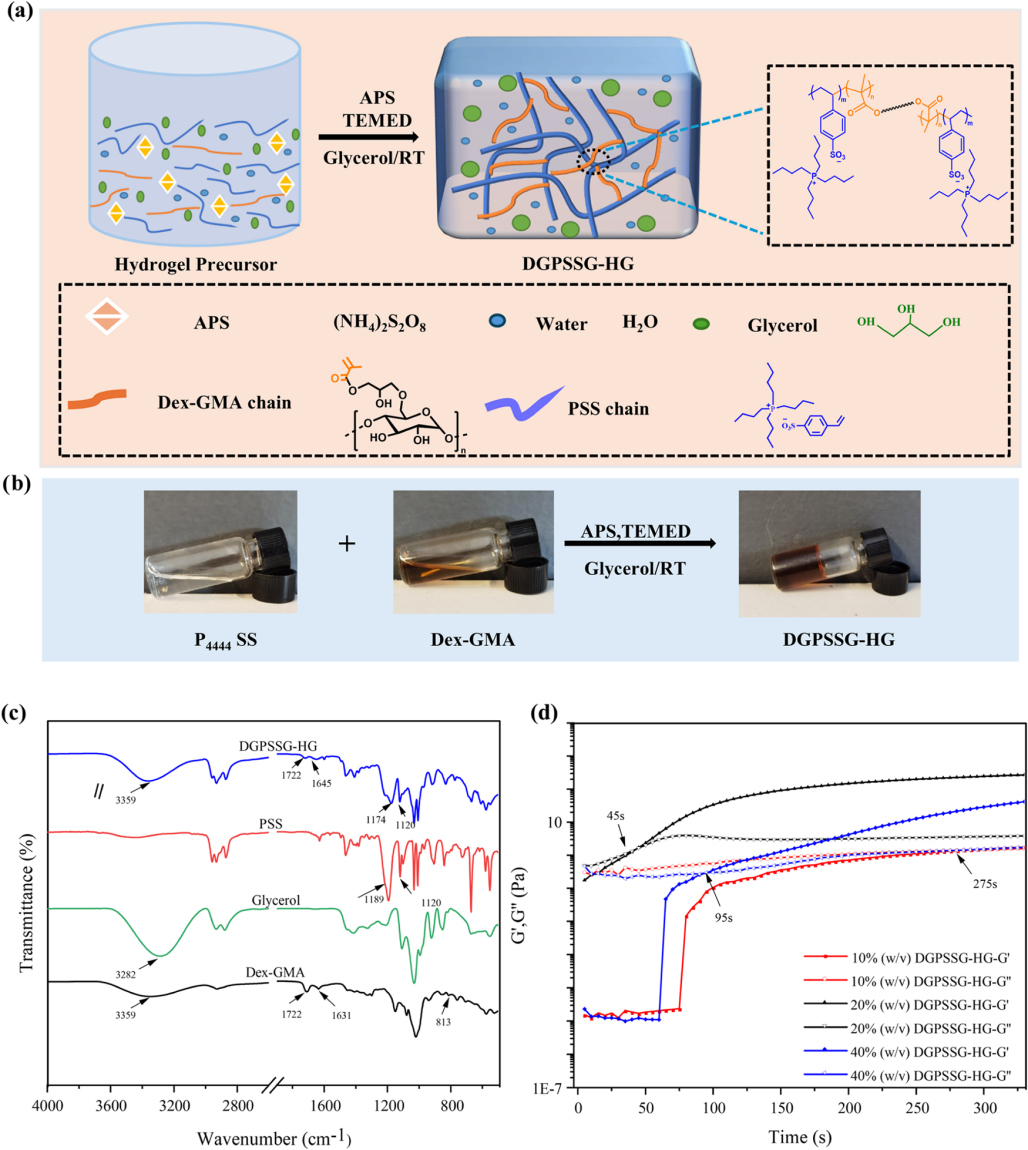
(a) Schematic illustration of the DGPSSG hydrogel design: components of the hydrogel mixture free-radical polymerization and cross-linking of Dex-GMA and PSS. (b) Photographs illustrating the preparation process of the DGPSSG hydrogel. (c) FTIR spectra of Dex-GMA, glycerol, PSS, and DGPSSG. (d) Gelation times of the DGPSSG hydrogels containing different concentrations of PSS.

### Gelation Time

To investigate the influence of the ionic liquid concentration on hydrogel gelation, specific amounts of Dex-GMA (0.06 g) were combined with varying concentrations of the ionic liquid PSS (10–40% (w/v)) at room temperature. The initial time (0 s) was defined as the point at which the precursor solution was injected into the rheometer. The gelation time was determined by the plateau phase of the storage modulus (*G*′) and the loss modulus (*G*″) of the hydrogel. Figure 1d shows that the precursor solutions of Dex-GMA and PSS initially remained in the liquid state. Rheological measurements revealed that *G*′ and *G*″ correspond to the elastic and viscous properties of the hydrogel, respectively, with the gelation (or phase transition) point indicated by the intersection of *G*′ and *G*″, where the elastic behavior (*G*′) surpassed the viscous behavior (*G*″). Notably, increasing the ionic liquid concentration significantly accelerated gelation, likely due to the greater availability of ethylene groups for cross-linking in free-radical polymerization. At a lower PSS concentration (10% w/v), gelation occurred at 275 s, whereas higher concentrations (20 and 40% w/v) reduced gelation times to 45 and 90 s, respectively. Interestingly, the 10 and 20% w/v samples exhibited a pronounced increase in *G*′, attributed to burst-like network formation and rapid cross-linking,^49^ resulting in a steep *G*′–time slope that reflects a high gelation rate. In contrast, the 40% w/v sample, despite its faster gelation onset, displayed a more gradual increase in *G*′, likely due to diffusion-limited polymerization caused by the higher viscosity of the concentrated system.^50^ This trend highlights the crucial role of PSS in gelation kinetics, as a higher ionic liquid concentration promotes faster polymerization and cross-linking, facilitating a more rapid transition from liquid to solid-like hydrogel.

### Thermal Stability

The thermal stability of the DGPSSG hydrogels was evaluated via thermogravimetric analysis (TGA) and derivative thermogravimetry (DTG) at a heating rate of 10 °C/min up to 700 °C. As shown in Figure 2 and detailed in Table S2 (in the Supporting Information), the hydrogels with different ionic liquid concentrations (10, 20, and 40% w/v) exhibited a multistage weight loss pattern. The TGA curves (Figure 2a) revealed two main weight loss steps: an initial loss below 100 °C due to water evaporation and a subsequent loss between 200 and 500 °C associated with thermal decomposition. At a residual weight fraction of 70%, the degradation temperatures were 218.20, 247.10, and 420.30 °C for the 10, 20, and 40% (w/v) hydrogels, respectively, with the 40% (w/v) hydrogel demonstrating the highest thermal stability. A similar trend was observed at 40% residual weight fraction. DTG analysis (Figure 2b) further revealed maximum decomposition temperatures of 454.00, 454.03, and 454.07 °C for the 10, 20, and 40% (w/v) hydrogels, respectively. These results demonstrate that increasing the ionic liquid content enhances the thermal stability of the hydrogel. This improvement is attributed to the role of the ionic liquid in strengthening cross-linking and promoting ionic interactions within the covalently cross-linked hydrogel network. These interactions stabilize the structure, increasing its resistance to thermal degradation.

**Figure 2:**
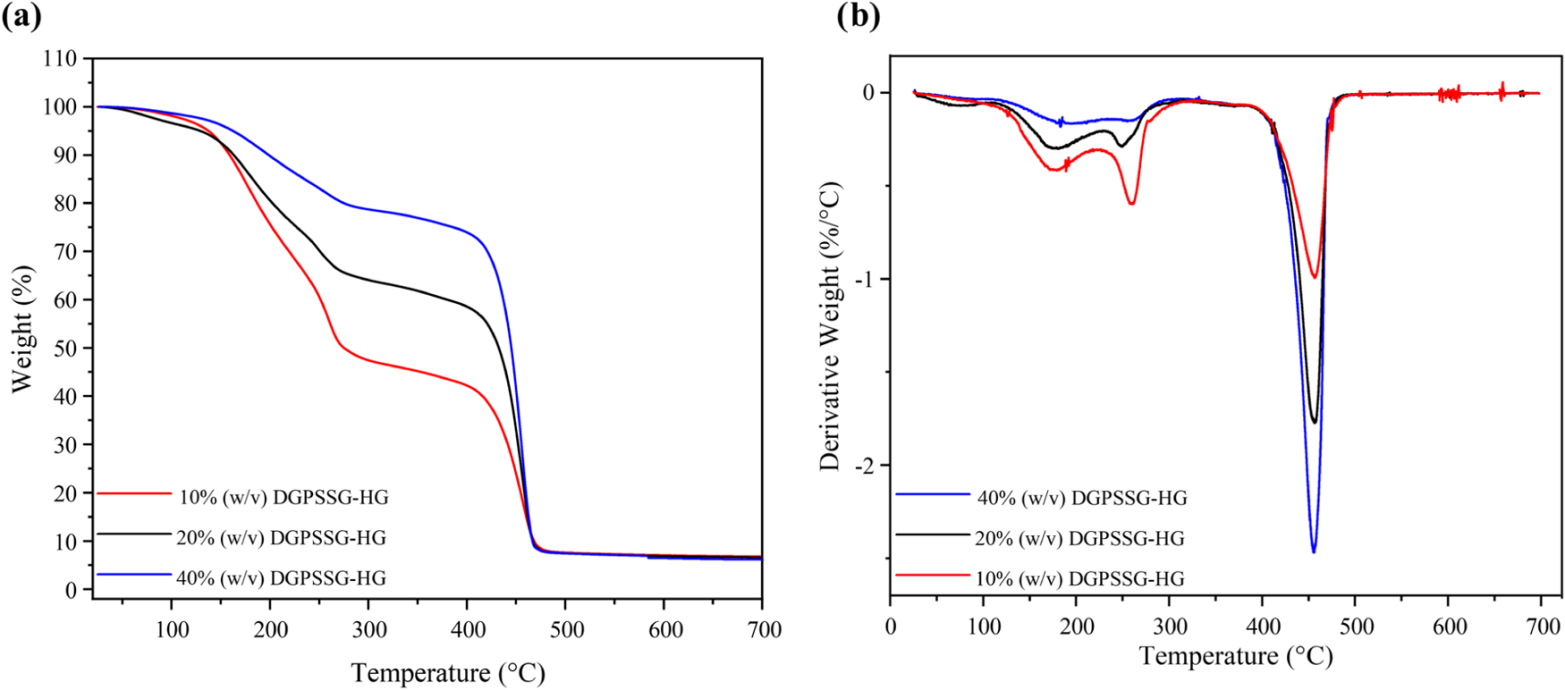
(a) TGA and (b) DTG analyses of the DGPSSG hydrogels containing different concentrations of PSS.

### Rheological Properties

The stiffness of the hydrogel is primarily provided by covalent bonds in the Dex-GMA-PSS network, whereas glycerol reinforces the structure through hydrogen bonding with Dex-GMA and PSS. This combination prevents rupture under stress while maintaining the elasticity of the hydrogel (Figure 3a). Additionally, rheological tests were conducted to evaluate the viscoelastic properties of the DGPSSG hydrogels, as shown in Figure 3b, by comparing the hydrogels with and without glycerol. The results demonstrated that the storage modulus (*G*′) was consistently greater than the loss modulus (*G*″), indicating predominantly elastic behavior.^51^ This enhanced elasticity is more pronounced in the DGPSSG hydrogels compared to the DGPSS hydrogels without glycerol, resulting in a significant disparity (*G*′ > *G*″). During the frequency sweep test, the storage modulus of the 20% (w/v) DGPSSG hydrogel increased from 140 to 178 Pa over the frequency range of 1–10 s^–1^. Both *G*′ and *G*″ exhibited nonlinear behavior at elevated frequencies, suggesting complex viscoelastic responses under these conditions. Compared with the loss modulus, the hydrogels DGPSS and DGPSSG have a higher storage modulus in the lower frequency range, indicating that the gel state is dominant. When glycerol was added to the hydrogel, it became more elastic, as indicated by the larger *G*′ > *G*″ ratio (∼7.48) of the DGPSSG hydrogel than the higher *G*′ > *G*‴ ratio (∼3.11) of the DGPSS hydrogel. The same relationship is then observed in separate DGPSS hydrogels with different ionic liquid contents (Figure S4a,b in the Supporting Information). This consistency indicates that both the ionic liquid concentration and glycerol content synergistically contribute to the mechanical properties of the hydrogel. The inclusion of glycerol not only reinforces the hydrogel network but also enables better stress dissipation, making the hydrogel more robust.

**Figure 3:**
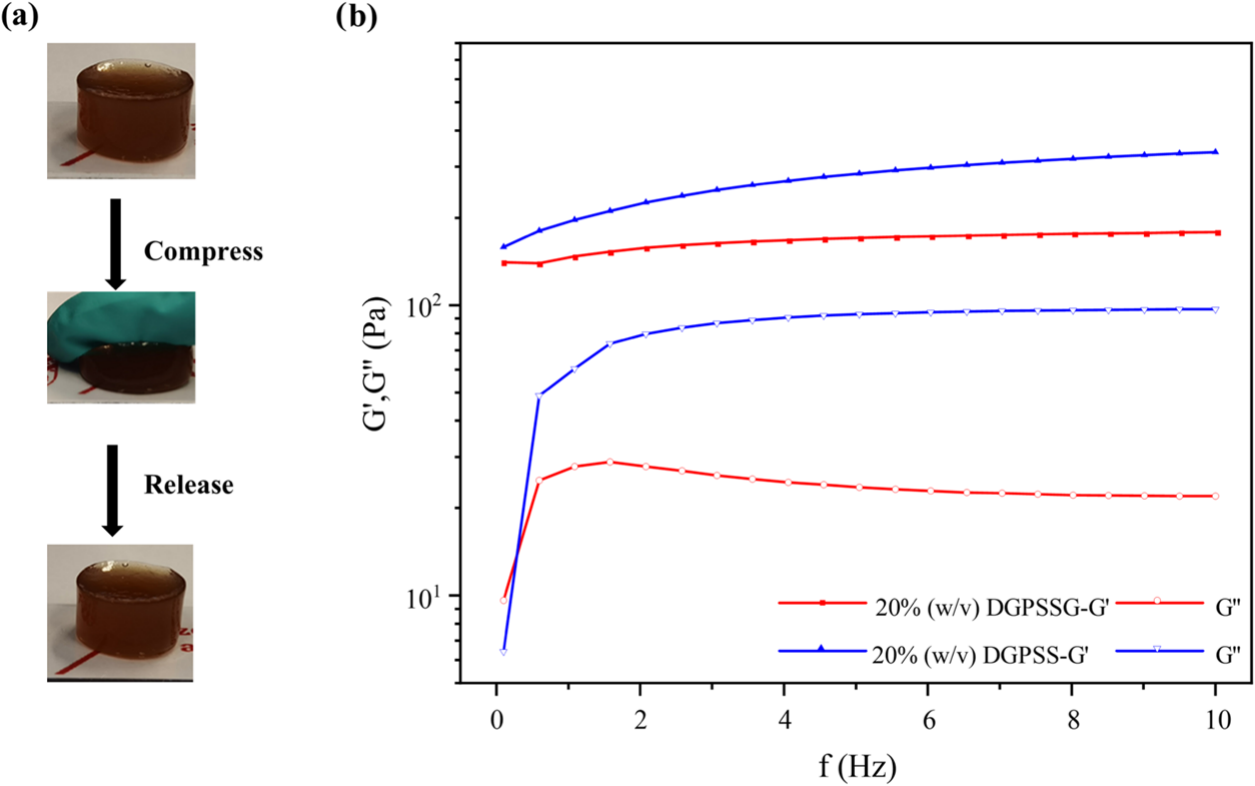
(a) Photographs of the compression performance of the DGPSSG hydrogel. (b) Storage modulus *G*′ and loss modulus *G*″ of the DGPSSG and DGPSS hydrogels versus frequency.

### Swelling Ratio and Water Retention Testing

The swelling ratio of the hydrogels is a critical indicator of their capacity to absorb water. As illustrated in Figure 4a, the swelling behavior of the DGPSSG hydrogels was monitored over time, revealing that the swelling ratios for all three hydrogel formulations reached equilibrium after approximately 2.5 h (150 min). This stability underscores the robustness and sustained water retention capability of the DGPSSG hydrogels. Among the formulations, the 40% DGPSSG hydrogel resulted in the highest swelling ratio, with a volume increase of nearly 4 times the initial volume, whereas the 10% (w/v) DGPSSG hydrogel resulted in the lowest swelling ratio, approximately double the initial volume. The superior water absorption capacity of the 40% (w/v) DGPSSG sample can be attributed to several key factors. First, the balance between hydrophilic and hydrophobic interactions shifts as the ionic liquid content increases, enhancing the hydrophilicity of the hydrogel. Ionic liquids generally exhibit a high affinity for water, allowing for the attraction and retention of a greater number of water molecules within the hydrogel network.^52^ Second, the increase in the ionic liquid content intensifies the electrostatic repulsion among ions in the hydrogel structure, leading to a more expanded or loosely arranged network that facilitates easier water penetration.^53^ Finally, the osmotic pressure difference between the interior of the hydrogel and the surrounding solution promotes water absorption to equalize the ion concentration gradient, further enhancing the swelling capacity. Collectively, these factors contribute to the elevated swelling ratios observed in ionic liquid hydrogels with higher ionic liquid contents.^54^ After 4.7 h (284 min), the swelling ratio of the 40% (w/v) DGPSSG sample continued to increase steadily, whereas the swelling ratios of the 20 and 10% (w/v) DGPSSG hydrogels remained stable, indicating the structural integrity of the DGPSSG hydrogels. This stability mitigates the risk of bursting associated with structural breakdown. Consequently, the incorporation of a small amount of ionic liquid into Dex-GMA significantly enhances the swelling capability of the hydrogel. Figure 4b further illustrates the water retention capabilities of the hydrogel components. Despite a steady decrease in water content over the first 2 days, the hydrogel retained more than 80% moisture. However, moisture diffusion over time slightly reduced the moisture retention. After 6 days, the water content stabilized at 75%, demonstrating the excellent water retention ability of the hydrogel. This is likely due to the increased water retention rates observed in ionic hydrogels with higher ionic liquid contents, which stem from increased hydrophilicity, increased electrostatic repulsion between ions, and decreased cross-linking density. Together, these factors expand the hydrogel network, facilitating improved water absorption and retention while balancing the osmotic pressure differences within the gel structure. These combined effects contribute to the hydrogel’s ability to maintain moisture over extended periods, making it suitable for applications in environments requiring prolonged hydration and stability.

**Figure 4:**
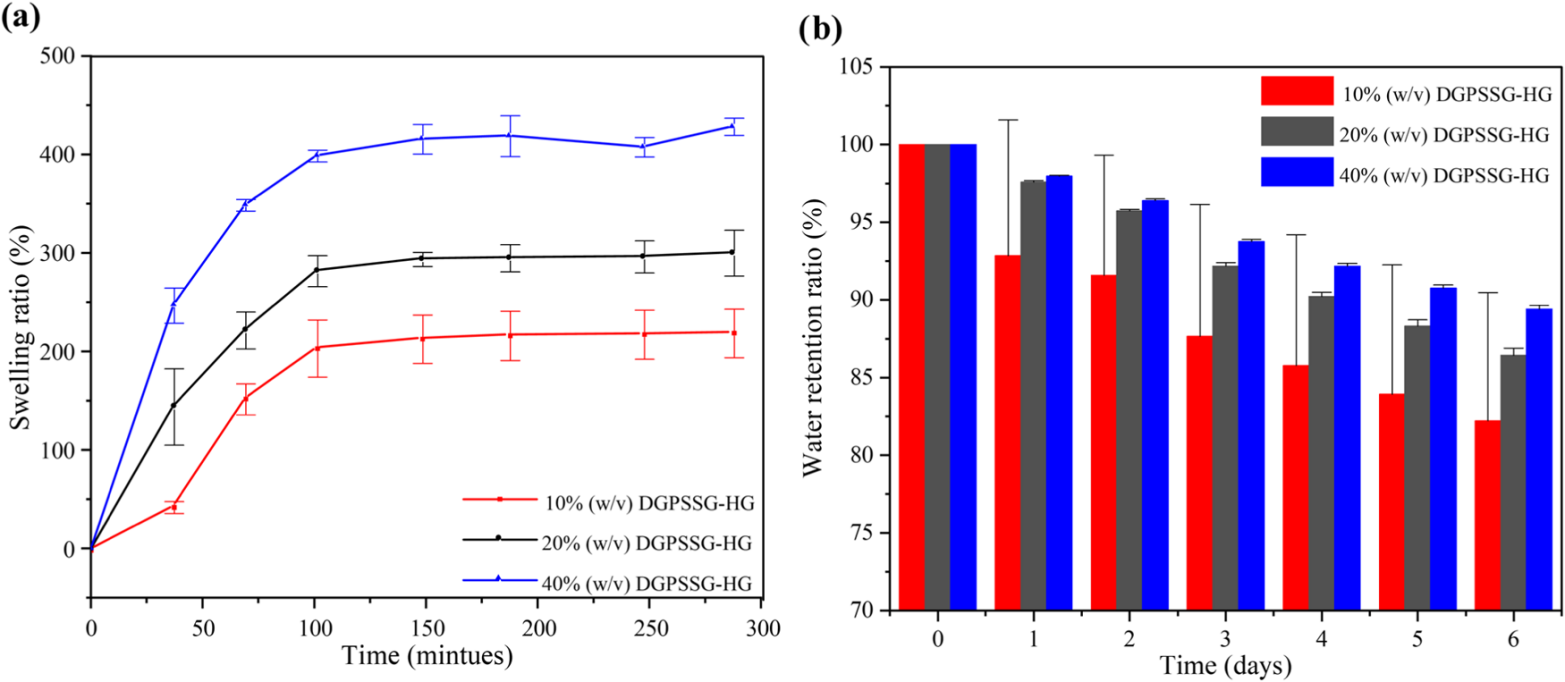
(a) Swelling ratios and (b) water retention rates of hydrogels with different ionic liquid contents.

### Antifreezing Properties

The high water content of the hydrogels renders their mechanical flexibility highly sensitive to temperature fluctuations. At low temperatures, hydrogels lose flexibility as the solvent (water) solidifies. The incorporation of glycerol effectively lowers the freezing point of water, thereby enhancing the antifreeze properties of the hydrogels.^55^ Creep-recovery tests were conducted to evaluate the antifreezing mechanical behavior of DGPSSG hydrogels with varying glycerol-to-ionic liquid (IL) ratios.^56^ Samples were subjected to gradually increasing stress up to 5 Pa over 300 s, followed by immediate stress removal. All tests were performed at controlled temperatures between −20 and 20 °C. Combined creep-recovery and rheological analyses demonstrated that hydrogels with a 1:3 glycerol-to-IL ratio exhibited the highest elastic recovery and retained flexibility even at −20 °C, confirming their excellent antifreeze performance (Figure S5a–d).To evaluate the antifreeze effect of glycerol on the hydrogel matrix with a 1:3 glycerol-to-IL ratio, the hydrogel samples, both with and without glycerol (DGPSSG and DGPSS), were stored together at −20 °C for 4 h. Figure 5a shows that various concentrations of the ionic liquid hydrogel without glycerol can completely freeze into a hard solid. In contrast, when glycerol is used as an antifreeze agent, the DGPSSG hydrogel remains soft and can retain its initial shape. The results were similar to those shown in Figure 5b; the DGPSS hydrogel was completely frozen and was extremely hard to compress. The DGPSSG hydrogel retains its softness and flexibility and can withstand compression at −20 °C without fracturing, in contrast to the DGPSS hydrogel (Supporting Videos S1 and S2). This proves the usability of the hydrogel at subzero temperatures. In the DSC curve shown in Figure 5c, distinct exothermic peaks appeared at −21.8, −26.6, and −24.6 °C in the DGPSS hydrogels without glycerol, indicating significant water crystallization under these conditions. Upon the addition of glycerol, the exothermic peak shifted to −45.2 °C (10% (w/v) DGPSSG-HG), indicating that glycerol effectively lowered the freezing point of water. Notably, when the ionic liquid content was increased to 20% (w/v) and 40% (w/v), the exothermic peaks completely disappeared, demonstrating that higher concentrations of ionic liquid combined with glycerol can further reduce the freezing point or even entirely inhibit ice formation. This enhanced antifreezing ability results from a synergistic effect: ionic liquids (ILs) consist of cations and anions with multiple hydrogen bond donor and acceptor sites, which can form strong hydrogen bonds and electrostatic interactions with water molecules.^57^ These interactions restrict the mobility and rearrangement of water molecules, hinder ice nucleation and crystal growth, and consequently lower the freezing point of the hydrogel.^58^ While the hydroxyl groups in glycerol form hydrogen bonds with water molecules, disrupting their orderly arrangement and thus preventing ice crystal formation.^59–61^ A similar phenomenon was observed in rheological tests conducted from −30 to 20 °C (Figure 5d). At temperatures below −7 °C, the *G*′ value of the 10% (w/v) DGPSS hydrogel increased sharply, indicating the onset of ice crystallization and a transition to a solid-like state. However, with the introduction of ionic liquid, this behavior was mitigated; the *G*′ values for the 20 and 40% (w/v) DGPSS hydrogels increased significantly only below −10 °C. In contrast, the DGPSSG hydrogels exhibited stable *G*′ values across the tested temperature range (from 20 to −30 °C), indicating that glycerol aids in maintaining the hydrogel’s flexibility at subzero temperatures.

**Figure 5:**
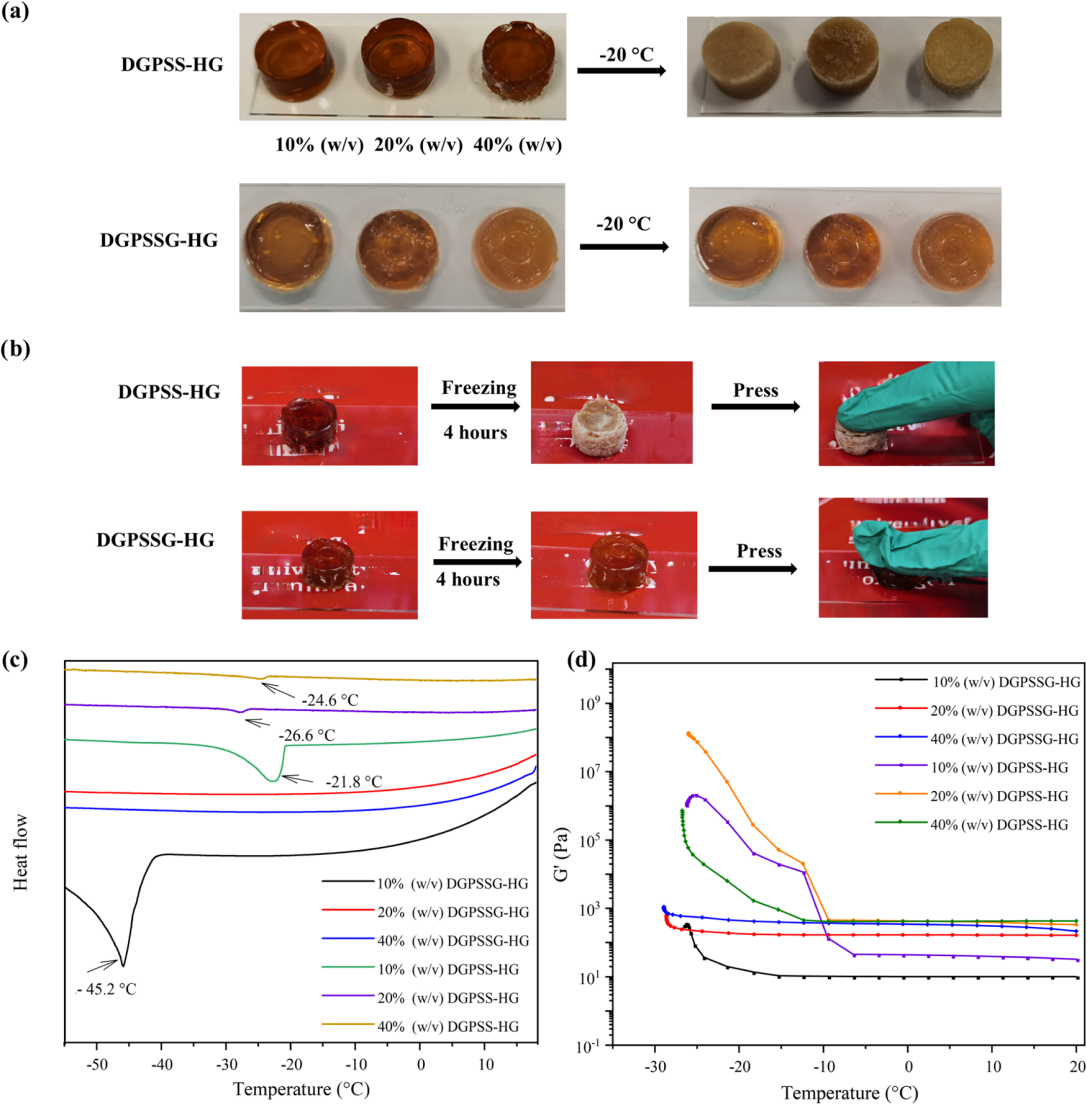
Antifreezing properties of the DGPSSG hydrogel. (a) Photographs of the DGPSS and DGPSSG hydrogels after 4 h of freezing at −20 °C. (b) Photographs of the DGPSS hydrogel and DGPSSG hydrogel compression after 4 h of freezing at −20 °C. (c) DSC thermograms of the DGPSS hydrogel and the DGPSSG hydrogel. (d) Storage modulus (*G*′) of the DGPSSG and DGPSS hydrogels during a temperature sweep at a constant shear strain of 0.1% and an angular frequency of 1 Hz.

### Conductivity Test

Furthermore, the antifreezing ability of DGPSSG related to its conductivity with glycerol was also explored (Figure 6a,b), and as the temperature changed from 20 to −20 °C, the conductivities of the different samples with and without glycerol greatly differed (Figure 6a). The stable conductivity was visualized by allowing the hydrogel to be refrigerated at different temperatures as a conductor connected in a closed circuit to light up the LED (Figure 6a). The DGPSS hydrogels lose the ability to conduct electrons at subzero temperatures. In contrast, when DGPSSG hydrogels, including glycerol, are used, LEDs can be lit successfully. The stability of the conductivity was assessed by measuring the electrical conductivity, which fluctuated between 0.054 ± 0.006 and 0.0207 ± 0.0010 μS cm^–1^ for the 20% (w/v) DGPSSG hydrogel as the temperature decreased from 20 to −20 °C (Figure 6b). In contrast, the conductivity of the DGPSS hydrogel without glycerol decreased rapidly under the same conditions. Owing to its remarkable antifreezing property, the conductivity of DGPSSG remained relatively stable even at −20 °C (Figure S6 in the Supporting Information). The reason for this outstanding antifreezing property is that glycerol can form many strong hydrogen bond clusters with water and disturb the formation of hydrogen bonds among water molecules, which mainly inhibits the freezing of the remaining water in the DGPSSG hydrogel networks.^62^

**Figure 6:**
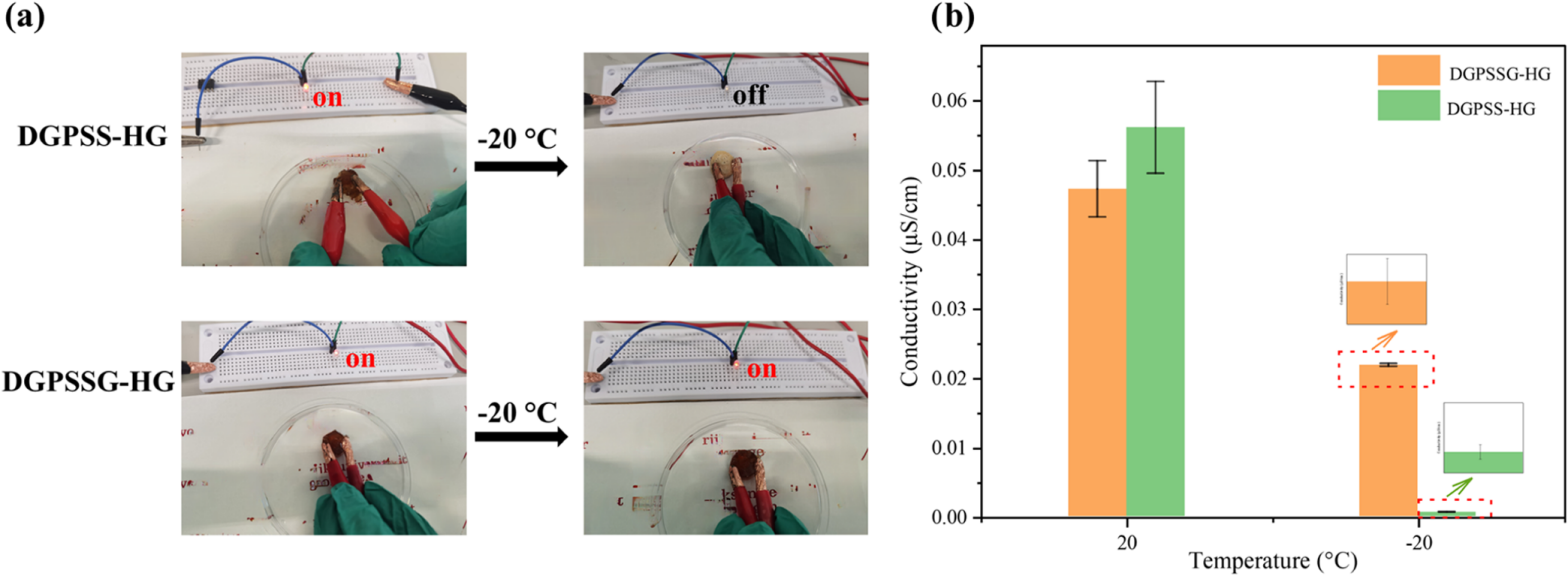
(a) Changes in the conductivity of the DGPSS-glycerol and DGPSS hydrogels with decreasing temperature from 20 to −20 °C. (b) Conductivity of the DGPSS-glycerol and DGPSS hydrogels at 20 and −20 °C.

### Thermoresponsive Properties of DGPSSG Hydrogel

The DGPSSG hydrogels can transition from transparent to opaque depending on the environmental temperature. Figure 7a,b shows the two different appearances of the ionic liquid solution and DGPSSG hydrogels at 20 and 60 °C, respectively. The optical microscopy images clearly demonstrate the thermoresponsive phase transition of the DGPSSG hydrogel^63^ (Figure 7c). Below the transition temperature, the hydrogel appears bright and transparent, allowing light to pass through with minimal scattering. In contrast, above the transition temperature, the hydrogel becomes opaque due to increased light scattering, resulting in a significantly darker image. Quantitative analysis using ImageJ revealed a marked decrease in the mean gray value after the phase transition, confirming the reduction in optical transmittance. For example, the 20% (w/v) DGPSSG hydrogel exhibits a high mean gray value (89.6) at low temperatures, indicating strong transparency. Upon heating above the transition temperature, the mean gray value decreases significantly to 47.9°, corresponding to a more opaque state. A similar trend is observed in two additional samples with different ionic liquid content (Figure S7c in the Supporting Information), providing both visual and quantitative evidence for the hydrogel’s thermoresponsive property. The SEM images of the DGPSSG hydrogel are shown in Figure 7d. To investigate the morphological changes before and after the phase transition, the hydrogels were maintained at 20 and 60 °C for 2 h before freeze-drying, thus ensuring full equilibration. After treatment, the samples were rapidly frozen in liquid nitrogen and subsequently lyophilized. At the lower temperatures, the hydrogel exhibited a uniform and well-distributed porous structure. In contrast, at the higher temperature, the pore structure was largely disrupted, and no intact porous network was observed, indicating significant structural rearrangement induced by the thermal phase transition.^64^ These properties arise from the formation of hydrogen bonds and electrostatic interactions between the polymer PSS chains and the surrounding solvent environment. This interaction leads to aggregation and phase separation, causing the hydrogel to reversibly transition from transparent to opaque (Figure 7e). Additionally, the DGPSSG hydrogel was subjected to 10 cycles at 20 and 60 °C, and an electrical circuit containing a light-emitting diode (LED) in series with our hydrogels was used to visually analyze the performance of the smart thermosensitive hydrogels (Figure S8a in the Supporting Information). Stability, reversibility, and continuity are crucial factors in evaluating the performance of thermosensitive materials. The DGPSSG hydrogel demonstrated stable and reversible resistance changes during continuous cooling–heating cycles (Figure S8b in the Supporting Information), highlighting its consistent and reliable thermal sensitivity. Figure 7f,g shows the temperature-dependent changes in the storage modulus (*G*′), loss modulus (*G*″), and loss factor (tanδ = *G″*/*G*′) of the DGPSSG hydrogels. These measurements provide insights into the thermoresponsive behavior of the hydrogels and their structural stability across different temperatures. Representative samples containing 10, 20, and 40% (w/v) PSS were studied to investigate how the ionic liquid content influences the thermal properties. As shown in Figure 7d, the hydrogels softened with increasing temperature, as indicated by a decrease in *G*′ and an increase in *G*″. This softening resulted from heat-induced polymer chain mobility and disrupted hydrogen bonding. Despite these changes, *G*′ consistently exceeded *G*″ between 20–70 °C, confirming the gel state. Near the phase transition, the *G*′ and *G*″ values are close, because of accelerated molecular motion and water absorption. Additionally, it is reasonable to assume that the higher ionic liquid concentration contributed to greater heat evolution during polymerization, leading to local temperature increases that exceeded the phase transition and resulted in inhomogeneity. Then, the thermoresponsive behavior was consistent with the LCST results, which were determined via the loss factor (tanδ). As shown in Figure 7g, the loss factor remained low below the phase transition temperature but increased significantly at its onset, indicating the LCST. The LCSTs for hydrogels with 10, 20, and 40% (w/v) PSS were 54.3, 32.9, and 28.6 °C, respectively. Additionally, time-dependent dynamic sweep cycles demonstrated the stable and reversible thermoresponsive behavior of the hydrogels. As shown in Figure 7h, both the storage modulus (*G*′) and loss modulus (*G*″) increased upon heating from 20 to 60 °C and decreased during cooling, indicating a stable and reversible thermoresponsive behavior.^65^ Similar results were observed in two additional samples (Figure S9a,b Supporting Information), confirming good consistency. Figure 7i shows that the equilibrium swelling ratio of the DGPSSG hydrogel is much lower at 60 °C than at 20 °C. This trend, also observed in other samples (Figure S7a,b in the Supporting Information), reflects the hydrogel’s LCST-type thermoresponsive behavior. Below the LCST, the hydrogel is highly hydrophilic, allowing more water absorption. Above the LCST, the polymer chains become more hydrophobic, leading to water expulsion and network shrinkage, thus reducing the swelling ratio.^27^ These results demonstrate that the ionic liquid concentration plays a key role in modulating the LCST and thermal behavior of the hydrogels. The DGPSSG hydrogels exhibited remarkably reversible changes in gel opacity induced by temperature changes (Figure S8c in the Supporting Information), emphasizing their potential for thermoresponsive applications.

**Figure 7:**
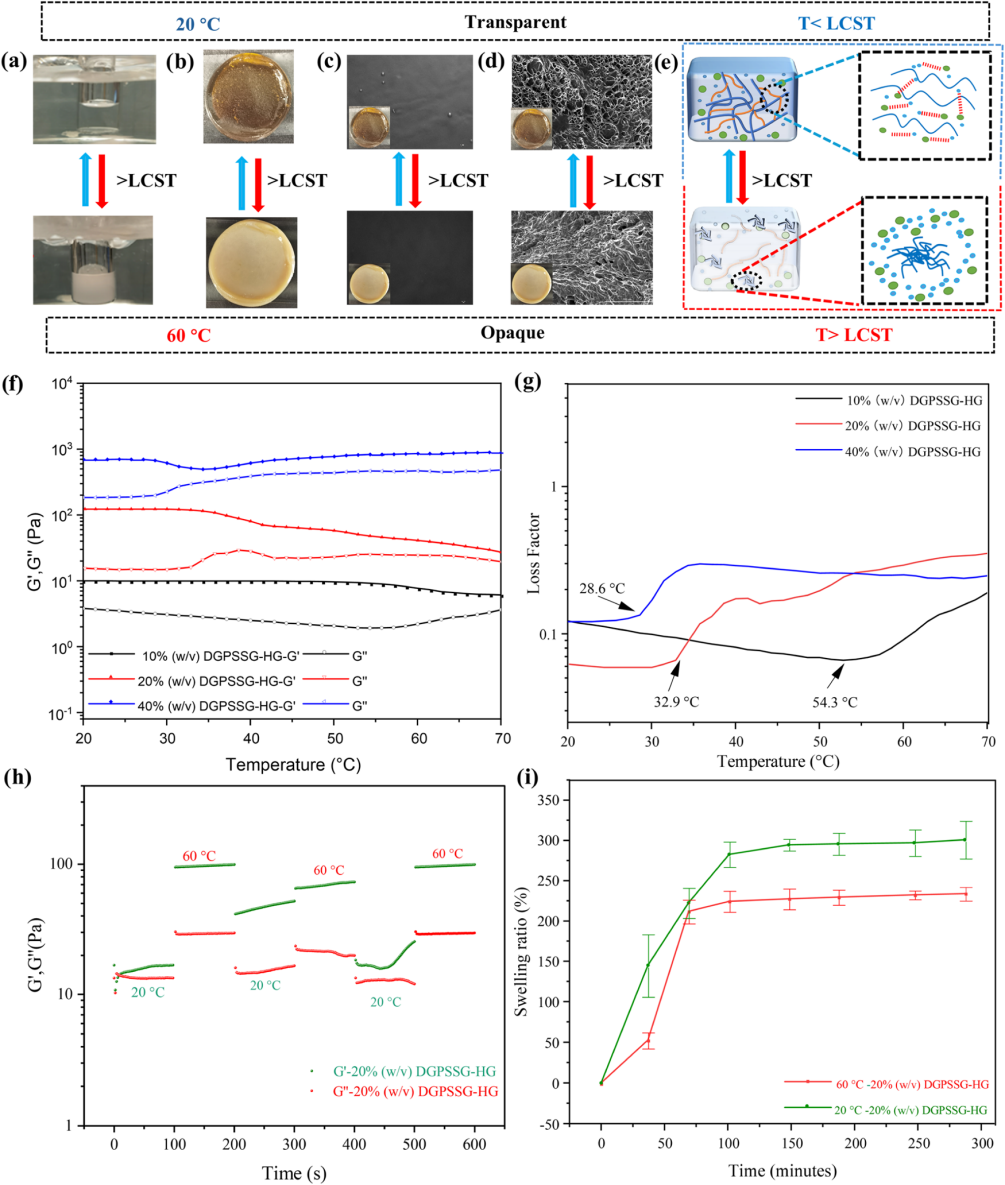
Thermoresponsive ability of hydrogels. (a) Illustrations of the LCST-type phase transition of a 20% (w/v) PSS solution taken at temperatures below and above the LCST. (b) Transparency of the smart hydrogel at temperatures below and above the LCST. (c, d) Optical microscopy and SEM of DGPSSG hydrogels at 20 and 60 °C. (e) Scheme of the temperature-responsive phase transformation mechanism. (f, g) Thermal scanning rheological observations of the different ionic liquid contents of the DGPSSG hydrogels. (f) Storage modulus (*G*′), loss modulus (*G*″), and (g) loss factor (tanδ). (h) Dynamic stepwise temperature amplitude tests of the DGPSSG hydrogel at different temperatures. (i) Swelling ratio of the smart hydrogel at different temperatures.

### Mechanical Properties of DGPSSG Hydrogels

To further evaluate the mechanical properties of the hydrogel, cylindrical samples (16 mm in diameter and 6 mm in thickness) were prepared and subjected to compression testing using a universal testing machine.^66^ As shown in Figure 8a, the hydrogel could withstand uniaxial compression up to 50% strain without fracturing and recovered its original shape upon load release, demonstrating excellent self-recovery behavior. The compressive properties of DGPSSG hydrogels with varying ionic liquid (IL) contents are presented in Figure 8b. As the IL content increased from 10 to 40% (w/v), the compressive strain exhibited a nonmonotonic trend, peaking at 20% (w/v). This suggests that the moderate incorporation of PSS enhances the flexibility and energy dissipation capacity of the hydrogel network. In contrast, excessive PSS (40% (w/v)) likely leads to an overly dense and rigid network structure, which restricts polymer chain mobility and reduces the deformability. Meanwhile, the compressive stress increased consistently with a higher PSS content, indicating improved mechanical strength due to enhanced ionic interactions and network reinforcement. Although primarily attributed to the IL content, the mechanical behavior may also be influenced by the network microstructure. The homogeneous free-radical polymerization conditions typically yield statistical copolymers, but microstructural effects cannot be excluded.^67^ Furthermore, cyclic loading–unloading tests were conducted at varying compressive strains (5, 10, 20, 30, 40, 50, and 60%) without intervals to evaluate the energy dissipation behavior of the hydrogel. Energy dissipation is typically represented by hysteresis loops in consecutive loading–unloading cycles, with the area of each loop corresponding to the energy dissipated per unit volume.^68^ As shown in Figure 8c, the hysteresis loop area increased with strain, and distinct loops were observed when the compressive strain exceeded 20%, indicating effective energy dissipation during large-strain deformation. The elastic behavior of the DGPSSG hydrogel was further investigated to assess its compression resilience and fatigue resistance, which are critical for repetitive pressure-sensing applications. As shown in Figure 8d, successive cyclic compression at 30% strain for 100 cycles was performed. The nearly overlapping curves in Figure 8e indicate excellent fatigue resistance and mechanical stability. This behavior is attributed to the presence of numerous reversible ionic and hydrogen bonds within the hydrogel network, which can rapidly reform after deformation, enabling the hydrogel to maintain structural integrity over repeated loading.^69^ Additionally, the hysteresis (toughness) and energy dissipation ratio were quantitatively calculated via numerical integration of the hysteresis loops^70^ (Figure 8f). The DGPSSG hydrogel exhibited a low energy dissipation ratio of approximately 20%, indicating its high elasticity and limited energy loss during deformation. Additionally, the antifatigue behavior of the antifreezing DGPSSG hydrogel was further examined through five consecutive cyclic loading–unloading tests at varying compressive strains of 30, 40, and 50% at 20 and −20 °C, without any intervals (Figure 8 g,h). During the unloading process, irreversible deformation was observed, particularly in the first cycle, accompanied by a slight decrease in the maximum stress over successive cycles. Nevertheless, the hydrogel still exhibited repeatable and consistent cyclic behavior. Furthermore, as shown in Figure S10, successive cyclic compression tests at 30% strain for 100 cycles at −20 °C demonstrated that the hydrogel maintained excellent elasticity, indicating outstanding fatigue resistance even under subzero conditions.

**Figure 8:**
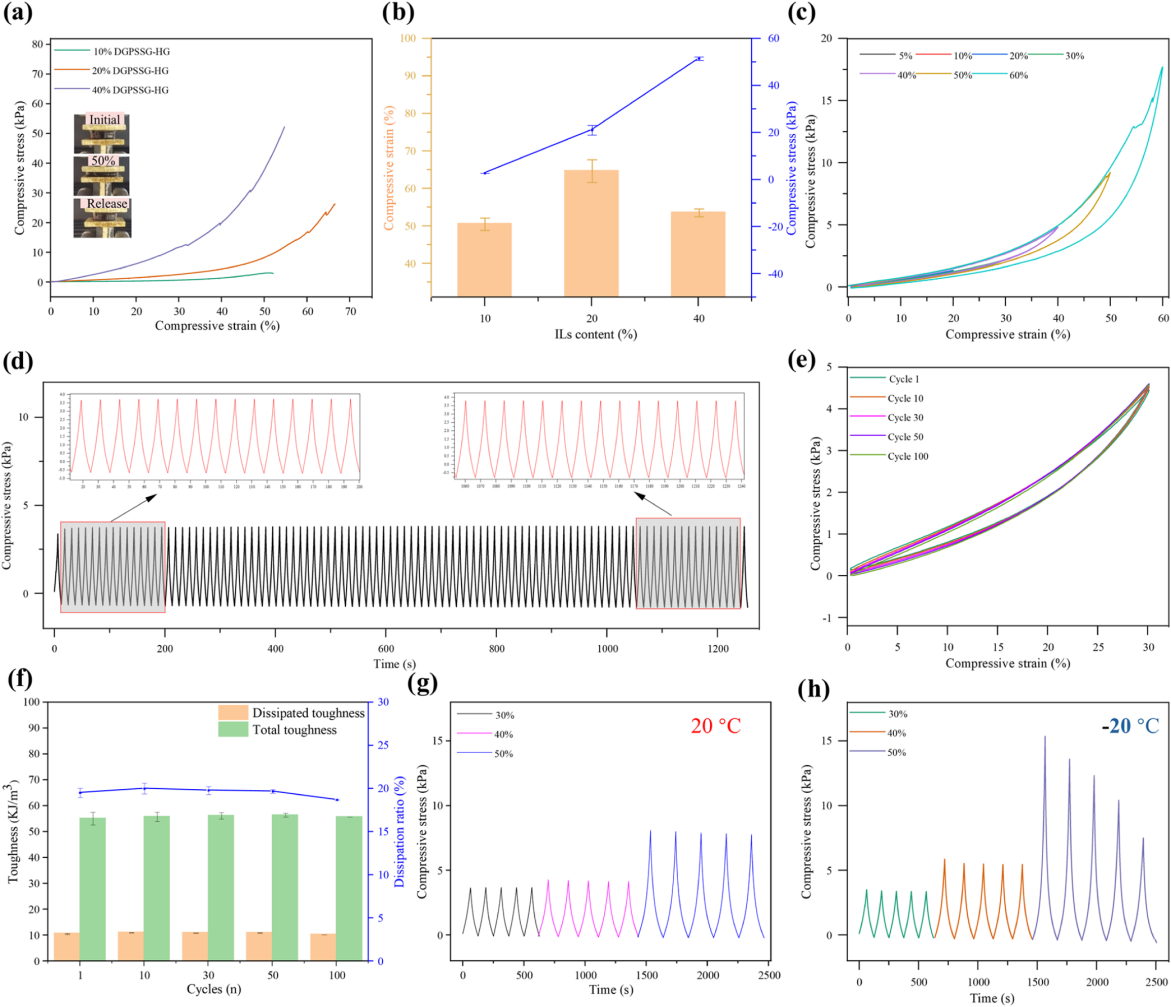
Compression tests of the prepared hydrogel: (a) compressive stress–strain curves of DGPSSG hydrogels with different IL contents. (b) Compression strain and stress of DGPSSG hydrogels with different IL contents. (c) sequential loading–unloading tests without an interval under varying strains of 5, 10, 20, 30, 40, 50, and 60%, respectively. (d) Compressive stress–strain curves of the hydrogel under 100 consecutive cycles at 30% strain. (e) Compressive stress–strain curves of the hydrogel under 100 consecutive cycles at 30% strain. (f) The corresponding total and dissipated toughness and energy dissipation ratio versus compressive cycle number. (g, h) Five successive loading–unloading cycles of DGPSSG hydrogel under different strains of 30, 40, and 50%, respectively, at 20 and −20 °C.

### Sensing Performance of DGPSSG Hydrogels

The sensing performance of the DGPSSG hydrogel under compressive strain was evaluated to assess its potential for pressure-sensing applications. As shown in the relationship between strain and relative resistance change ((*R* – *R*_0_)/*R*_0_, where *R*_0_ and *R* represent the initial and loaded resistances, respectively) was investigated. The gauge factor (GF), defined as GF = ((*R* – *R*_0_)/*R*_0_)/ε, is commonly used to quantify sensitivity.^71^ The DGPSSG hydrogel sensor exhibited GF values of −1.27576 in the 0–30% strain range and −4.36121 in the 30–60% range, indicating excellent strain sensitivity over a wide range (Figure 9a). Figure 9b,c illustrates the resistance change of the DGPSSG hydrogel sensor under varying cyclic compressive strains at a loading rate of 2 mm·min^–1^. Notably, the sensor operated reliably within a strain range of 2 to 40%, demonstrating excellent reliability and stability. Furthermore, when the compression rate was varied from 1 to 20 mm·min^–1^, the sensor maintained consistent resistance changes under 10% strain (Figure 9d), highlighting its rate-independent sensing behavior. Additionally, the sensor’s resistance change remained stable over 100 consecutive cycles at 30% compressive strain at a loading rate of 20 mm·min^–1^ (Figure 9e), confirming its durability and repeatability. To evaluate the sensor’s performance in an extreme environment, the sensor was also tested at −20 °C. Figure 9f shows that the hydrogel sensor exhibits stable resistance changes under a 20–40% compressive strain. Similarly, under a 30% compressive strain, the change in the resistance of the sensor showed excellent stability and repeatability, which can be attributed to the elasticity and rapid recoverability of the hydrogel (Figure 9g). These results confirm the hydrogel’s stable sensing performance and durability in ambient and low-temperature environments, thus establishing a solid foundation for its further development and practical application.

**Figure 9:**
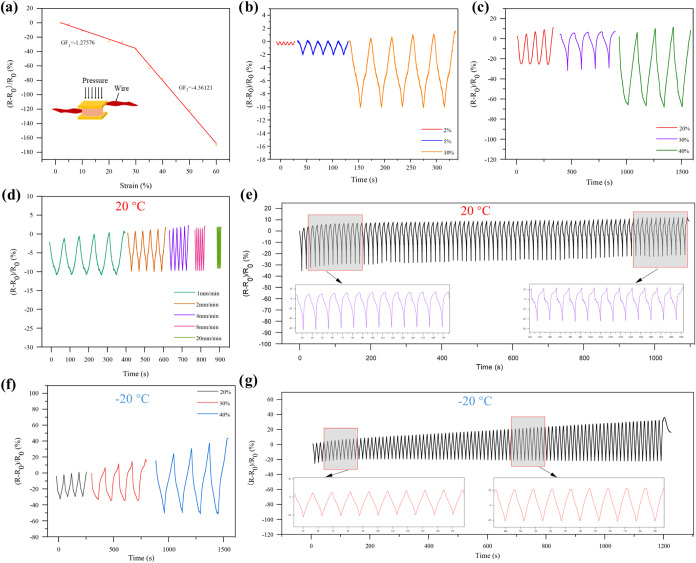
Sensing properties of DGPSSG hydrogel sensors: (a) The sensitivity of the DGPSSG hydrogel-based sensor. (b, c) Relative resistance changes in the DGPSSG hydrogel during small deformations (2, 5, and 10%) and high deformations (20, 30 and 40%). (d) Resistance variation in response to differing compression rates under 10% strain, (e) Relative resistance variation during cyclic compression testing over 100 cycles at 30% compressive strain at 20 °C. (f) Resistance variation in response to differing compression strain, at −20 °C, and (g) Relative resistance variation during cyclic compression testing over 100 cycles under 30% compressive strain at −20 °C.

### Binary Encoding, Encrypted Communication, and Real-Time Information Transmission of the DGPSSG Hydrogel

We employed the outstanding thermal responsiveness of the DGPSSG hydrogels to disguise and encrypt a wide range of secure communication. As shown in Figure 10a,b, covering the images (such as tulips) and drawings with the hydrogel allow us to view them only at room temperature (20 °C). However, as the temperature increased (60 °C), the hydrogel became opaque, and the patterns became unreadable. This reversible process allowed the patterns to reappear as the temperature decreased back to room temperature, with the hydrogel returning to its transparent state. Furthermore, the hydrogel serves as a visible temperature indicator of the temperature range of the surroundings and has the potential to monitor subjects where direct temperature measurement is not easy, such as fruit. As shown in Figure 10c, the square hydrogel, placed in an orange peel, transitions between transparent and opaque states on the basis of the surrounding temperature (20 or 60 °C). Additionally, the hydrogel effectively indicates the water temperature in a mug, as demonstrated in Figure S11. The DGPSSG hydrogel, capable of transitioning between transparent and opaque states by temperature control, offers the potential for information encoding and encryption. For binary encoding, the transparent Dex-GMA solution block represents binary “0”, whereas the thermally responsive DGPSSG hydrogel block represents binary “1”. These two precursor solutions were molded into 1 cm^3^ blocks. As shown in Figure 10d, these blocks can be programmed and assembled into sequences where each line corresponds to a letter in the binary code. Figure 10e,f shows the thermal responsive properties of the DGPSSG hydrogels. At 20 °C, the entire assembly appears transparent, but upon heating, the thermally responsive blocks become opaque, revealing encoded information such as “GEL” or “RUG”. This programmable encryption technique uses temperature control for concealing or revealing information. Our findings offer an alternative perspective for developing temperature-responsive materials with potential applications by establishing and setting a particular temperature for revealing and concealing information, which can be extended to information encryption and decryption.

**Figure 10:**
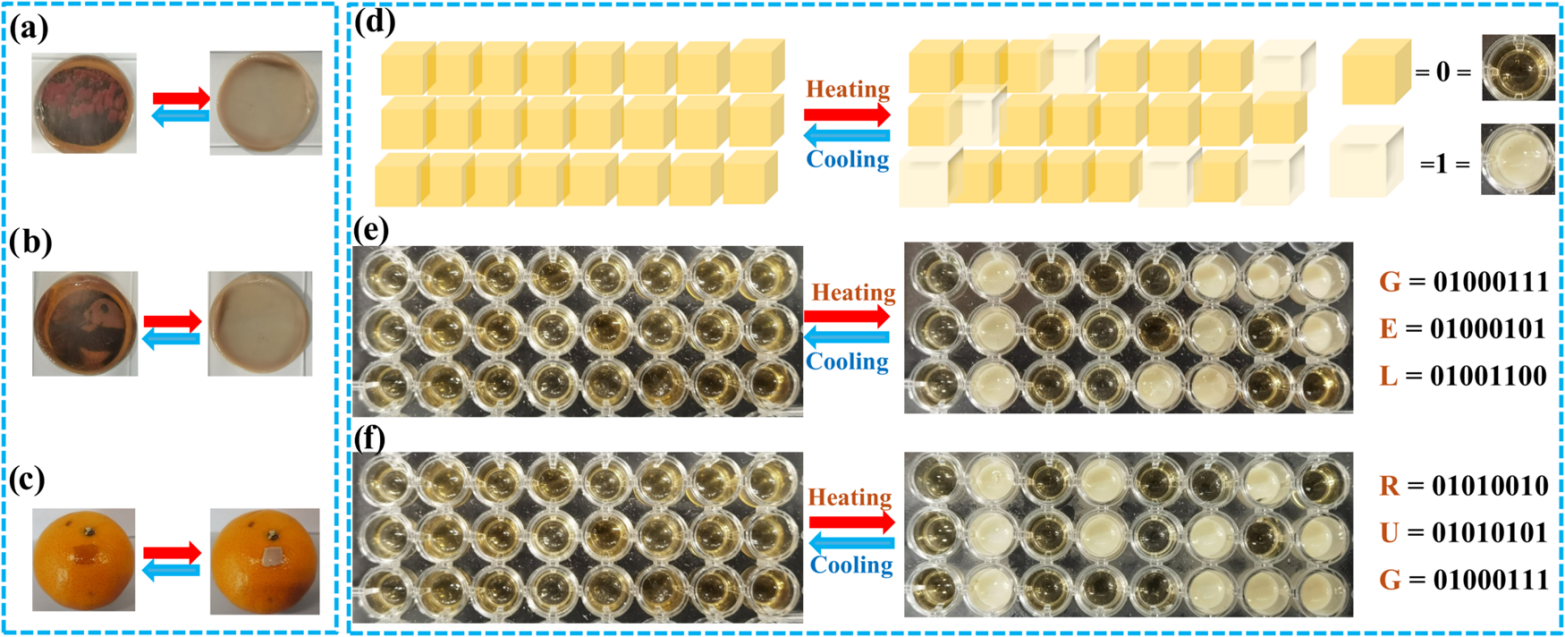
(a, b) DGPSSG hydrogel for anticounterfeiting and message encryption. (c) Temperature responsiveness of the DGPSSG hydrogel on orange peels. (d) Schematic diagram of binary encoding of a DGPSSG hydrogel. (e, f) Pictures of binary encoding of the DGPSSG hydrogel.

The Morse code, famous for its simplicity and efficiency, has been widely applied in fields such as information coding, aviation, and radio communication.^72^ It uses a combination of dots (.) and dashes (−) to represent letters of the alphabet (Figure 11a), enabling quick and accurate message transmission. In this study, inspired by the hydrogel’s ability to maintain elasticity under compression, elasticity was maintained. We developed a wearable communicator based on Morse code. Traditional hydrogels usually lose their mechanical properties in low-temperature environments, limiting their applications in cold conditions. However, our hydrogel can operate at both normal and low temperatures (Figure 11b). The communication mechanism defines a quick tap as “.” and a short tap as “-”, allowing messages to be crafted through deliberate tapping patterns (Figure 11c). As shown in Figure 11d–f, combining taps at two distinct speeds generates signals representing words such as “CALL,” “HELP,” and “RUG” at 20 °C. To evaluate its performance in cold environments, Morse code transmission was also conducted at −20 °C (Figure 11g–i). The sensor successfully transmitted encrypted messages, including “SOS”, “YES”, and “NO” demonstrating its potential for communication in extreme or emergency situations such as disaster relief or rescue operations.

**Figure 11:**
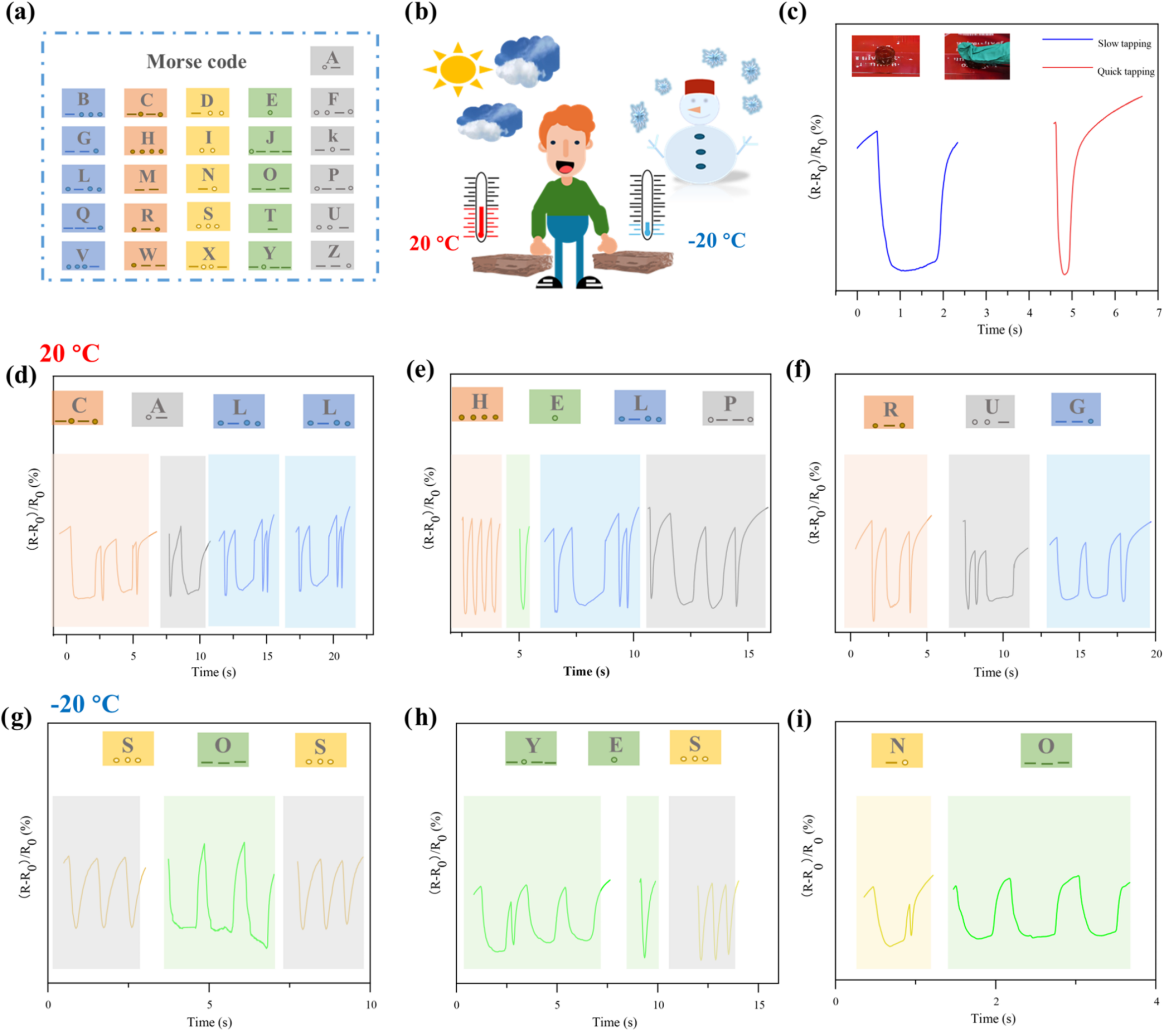
(a) Schematic illustration of the 26 Latin alphabet letters corresponding to Morse code. (b) Diagram of Morse code communication through the hydrogel sensor with messages sent at 20 and −20 °C. (c) The relative resistance changes when different speeds of tapping were used to record “dots” and “dashes” of the Morse code. The DGPSSG hydrogel sensor can accurately record Latin alphabet letters (“CALL”, “HELP” and “RUG”) (d, e, and f, respectively) at 20 °C and Latin alphabet letters (“SOS”, “YES” and “NO”) (g, h), and (i) at −20 °C through Morse code.

### Application of the DGPSSG Hydrogel as a Strain Sensor

To evaluate the possibility of using the DGPSSG hydrogel as a wearable sensor, we placed the hydrogel on different body parts and tested its electrochemical sensitivity during movement. Figure 12 shows several applications for sensing human motion, demonstrating the sensor’s potential in flexible electronic systems. The DGPSSG hydrogel exhibited excellent cycle stability. To track human emotion on the face, the resistive response signal in Figure 12a,b remained repeatable and constant in two different facial motions. Moreover, the hydrogel sensor can be used to monitor human activities. The resistive response signal, displayed in Figure 12c, remained steady and repeatable under continuous arm flexion and extension. When the change in the (*R* – *R*_0_)/*R*_0_ signal was measured at different angles, a strong positive correlation was observed between the angle and signal strength. The relative resistance remained constant at a given angle, and the signal completely recovered when the arm was straightened. Similarly, Figure 12d–f shows the sensor effectively tracked wrist flexion, finger bending, and leg motions with electrical signal movements exactly synchronized with body motion, demonstrating no lag. These results highlight the high sensitivity, stability, and suitability of the DGPSSG hydrogel-based wearable sensor for motion detection, indicating its potential as a reliable wearable sensor.

**Figure 12:**
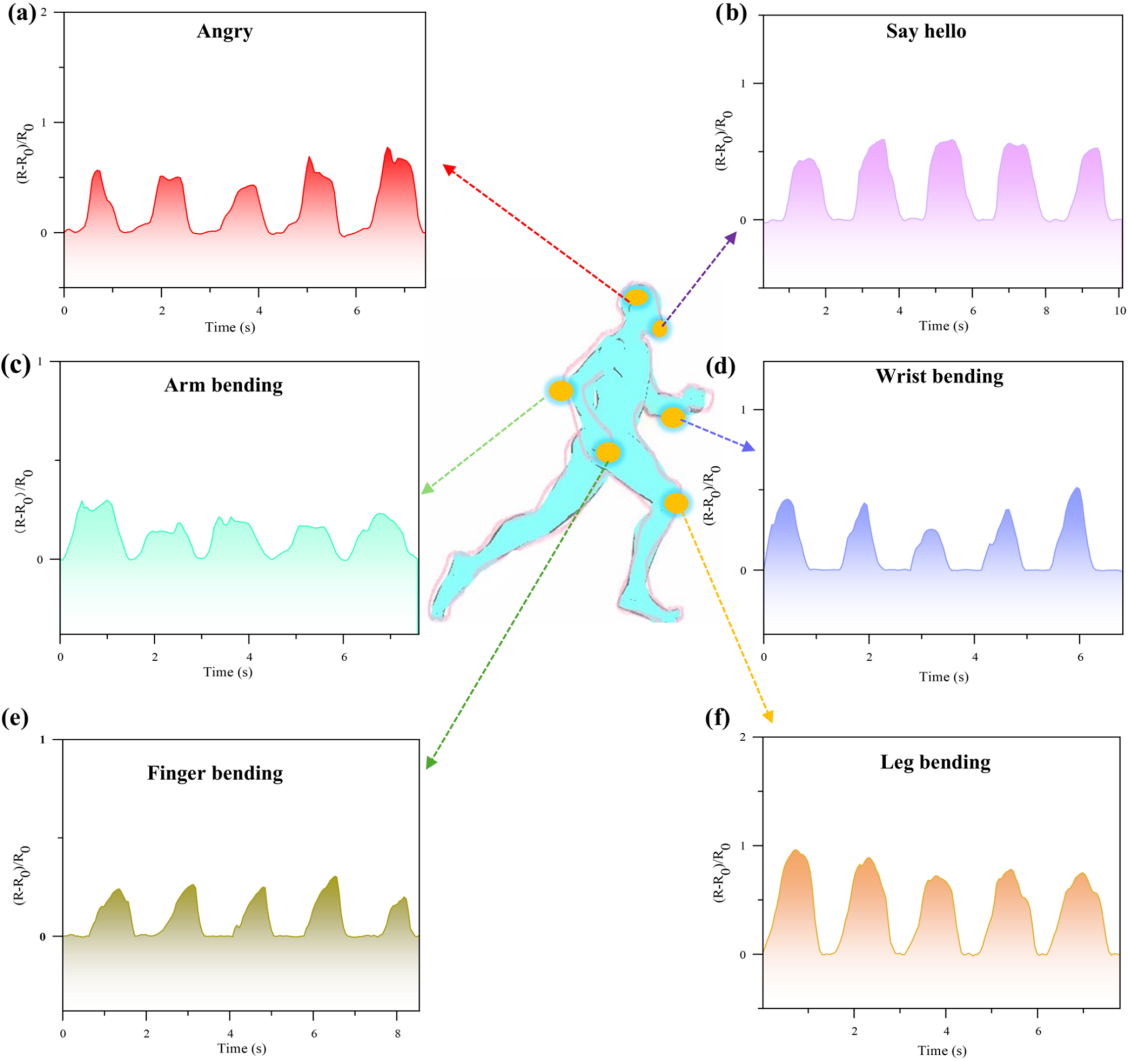
Human body motion detection of the DGPSSG hydrogel. Real-time monitoring of tiny human motions: frowning (a) and mouth movement (b) Relative resistance changes (RRCs) from arm motion angles of 0 to 90° (c), wrist motion (d), finger motion (e), and leg motion (f).

## Conclusions

In conclusion, we created a novel antifreezing, elastic, and temperature-sensitive hybrid DGPSSG hydrogel using a simple copolymerization process integrating temperature-responsive PSS (LCST-type) and glycidyl methacrylate-modified dextrin (Dex-GMA) in a glycerol–water solvent system. The resultant DGPSSG hydrogel demonstrates exceptional mechanical strength, remarkably low-temperature tolerance, and ionic conductivity, making it an attractive candidate for flexible sensor applications. The incorporation of PSS imparts ionic conductivity and tunable thermoresponsiveness (the LCST from 28.6 to 54.3 °C), while glycerol significantly improves the hydrogel’s antifreezing performance (down to −45.2 °C), enabling stable mechanical flexibility even at −20 °C. The DGPSSG hydrogel exhibits outstanding compressibility (from 5 to 60% strain), resilience, and long-term stability during cyclic mechanical deformation (over 100 cycles) through a wide range of temperatures (from −20 to 20 °C). It can be utilized as a wearable, flexible sensor capable of effectively monitoring subtle human motions (frowning and speaking) and joint movements. Furthermore, the sensor can detect human motions and transmit information in the form of Morse codes at room temperature and even at −20 °C. Notably, the DGPSSG hydrogel also shows a reversible temperature-induced transparency shift, transitioning from transparent to opaque (from 20 to 60 °C) above its LCST due to the hydrophilic to hydrophobic transition of PSS. This unique property has potential uses in multilevel optical encryption and decryption, enabling environment-responsive information concealment and display. Overall, this study proposes a simple and creative method for creating a multifunctional hydrogel with high sensitivity to both mechanical and temperature changes. The straightforward fabrication procedure, combined with excellent low-temperature performance, establishes the DGPSSG hydrogel as a promising multifunctional material for next-generation wearable electronics and sensing and secure communication technologies.

## Supplementary Material







## Data Availability

The authors confirm that the data supporting the findings of this study are available within the article and its Supporting Information.

## Notes

DGPSSG hydrogel retains its softness and flexibility and can withstand compression at −20 °C without fracturing (MP4)
Usability of the hydrogel at subzero temperatures (MP4)
Synthesis of PSS and Dex-GMA; FTIR spectra and ^1^H NMR spectra of dextrin and Dex-GMA; FTIR spectra and ^1^H NMR spectra of Na­[SS], [P_4444_]Br and PSS; compositions of the hydrogels (Table S1); the degradation temperatures of hydrogels (Table S2); storage modulus *G*′ and loss modulus *G*″ of the DGPSSG and DGPSS hydrogels versus frequency. Compositions of the hydrogels with different glycerol/ILs ratios (Table S3); the creep-recovery tests of the DGPSSG hydrogels; conductivity of the DGPSSG hydrogel at 20 and −20 °C; comparison of swelling ratios of the DGPSSG hydrogels at 20 and 60 °C; mean gray value of DGPSSG hydrogels with different ionic liquid contents at 20 and 60 °C; photographs and conductivity measurements of the DGPSSG hydrogel; dynamic stepwise temperature amplitude tests of DGPSSG hydrogel; compressive stress–strain curves of the hydrogel at −20 °C, and DGPSSG hydrogels for temperature sensors in a certain range (PDF)
