# Supplementary material for: Antifreezing and Temperature-Responsive Ionic Hydrogels with Applications in Encryption and Sensor Technologies
Source: ACS Appl Mater Interfaces. 2025 Jul 14;17(29):42303–20. doi: 10.1021/acsami.5c08600 (PMC12291081; doi:10.1021/acsami.5c08600)
Supplement: Supplementary file 3 [file am5c08600_si_003.pdf]

Supporting information

Antifreezing and Temperature-Responsive Ionic Hydrogels with Applications in Encryption and Sensor Technologies

*Xia Qiu[a,c#], Xiaolong He[b#], Kubra Kalayci[a,c], Paul Morandi[a,c], Petra Rudolf[b], Rudy Folkersma[c], Vincent S.D. Voet[c] and Katja Loos[*a]*

[a]Macromolecular Chemistry and New Polymeric Materials, Zernike Institute for Advanced Materials, University of Groningen, Nijenborgh 3, 9747 AG Groningen, The Netherlands

[b]Surfaces and Thin Films, Zernike Institute for Advanced Materials, University of Groningen, Nijenborgh 3, 9747 AG Groningen, The Netherlands

[c]Circular Plastics, Academy Technology & Innovation, NHL Stenden University of Applied Sciences, Van Schaikweg 94, 7811 KL Emmen, The Netherlands

*Corresponding author for this work: k.u.loos@rug.nl

[#]These authors contributed equally to this work.

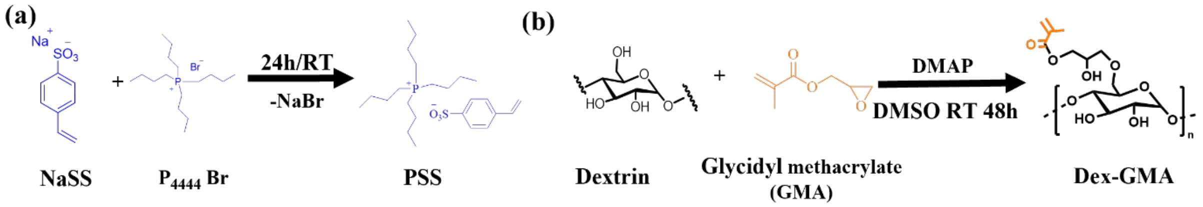

Figure S1. (a) Synthesis route to PSS through an anion exchange reaction. (b) Reaction scheme for the synthesis of Dex-GMA.

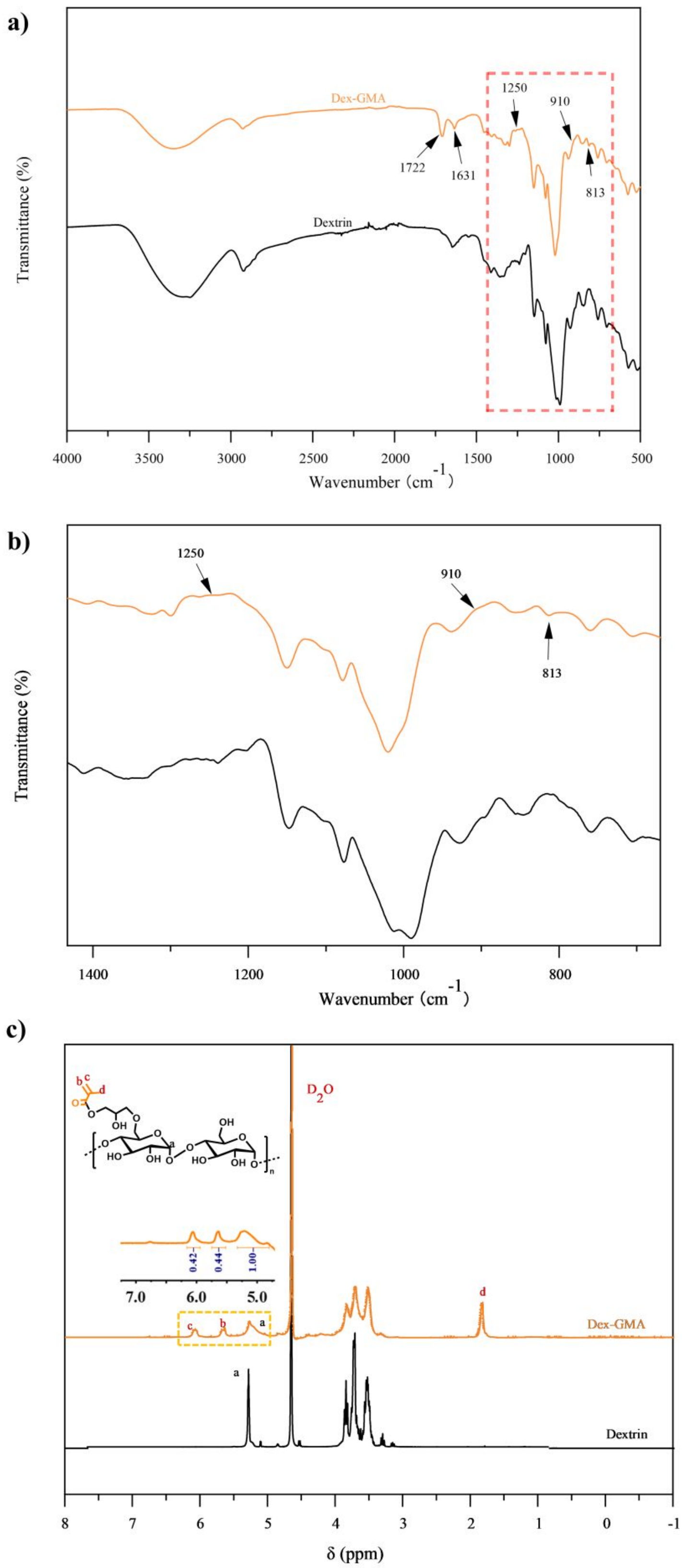

Figure S2. (a) FTIR spectra of dextrin and Dex-GMA. (b) Enlarged FTIR spectra of dextrin and Dex-GMA from 1430 cm^{-1} to 670 cm^{-1}. (c) ^{1}H NMR spectra of dextrin and Dex-GMA in D_2O

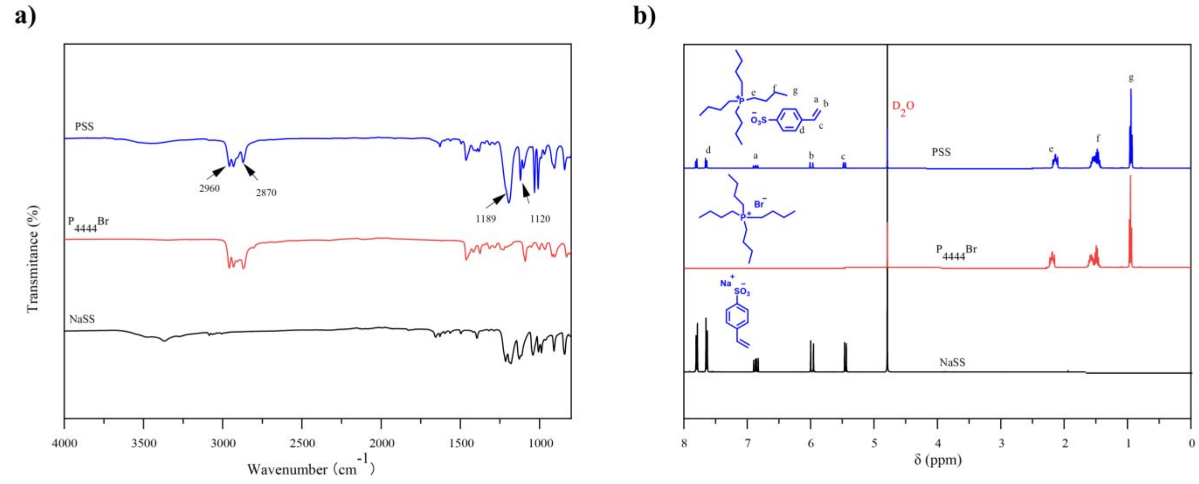

Figure S3. (a) FTIR spectra of Na[SS], $[P_{4444}]Br$ and PSS. (b) ^1H NMR spectra of Na[SS], $[P_{4444}]Br$ and PSS in D_2O.

Table S1. Compositions of the hydrogels.

Name<br>Abbreviation	Dex-GMA<br>(mg)	PSS content<br>(w/v)	H2O/Glycerol<br>(w/w)	APS<br>(mg/mL)	TEMED<br>μL/mL
10% (w/v) DGPSS-HG	60	10%	3:0	20	24
20% (w/v) DGPSS-HG	60	20%	3:0	20	24
40% (w/v) DGPSS-HG	60	40%	3:0	20	24
10% (w/v) DGPSSG-HG	60	10%	1:3	20	24
20% (w/v) DGPSSG-HG	60	20%	1:3	20	24
40% (w/v) DGPSSG-HG	60	40%	1:3	20	24

Table S2: The degradation temperatures of hydrogels with 10%, 20%, and 40% (w/v) of ionic liquid at different weight loss rates.

Samples	Degradation temperature at the residual weight of 70% (°C)	Degradation temperature at the residual weight of 40% (°C)	The maximum degradation peak (°C)
10% (w/v) DGPSS-HG	218.20	411.70	454.00
20% (w/v) DGPSS-HG	247.10	444.40	454.03
40% (w/v) DGPSS-HG	420.30	450.70	454.07

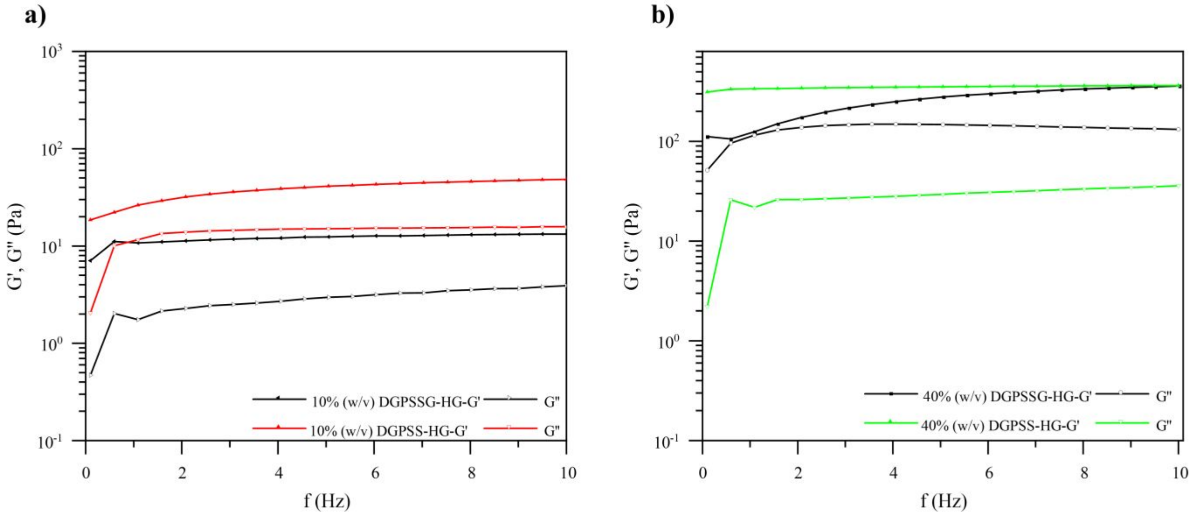

Figure S4. (a) and (b) Storage modulus G′ and loss modulus G″ of the DGPSSG and DGPSS hydrogels with different ionic liquid contents versus frequency

Table S3. Compositions of the hydrogels with different glycerol/ILs ratios under 20% (w/v) PSS system.

Name<br>Abbreviation	Dex-GMA<br>(mg)	PSS content<br>(w/v)	Glycerol/H2O<br>(w/w)	APS<br>(mg/mL)	TEMED<br>μL/mL
20% (w/v) DGPSSG-HG-1	60	20%	1:1	20	24
20% (w/v) DGPSSG-HG-2	60	20%	1:2	20	24
20% (w/v) DGPSSG-HG-3	60	20%	1:3	20	24
20% (w/v) DGPSSG-HG-4	60	20%	1:4	20	24
20% (w/v) DGPSSG-HG-5	60	20%	1:8	20	24

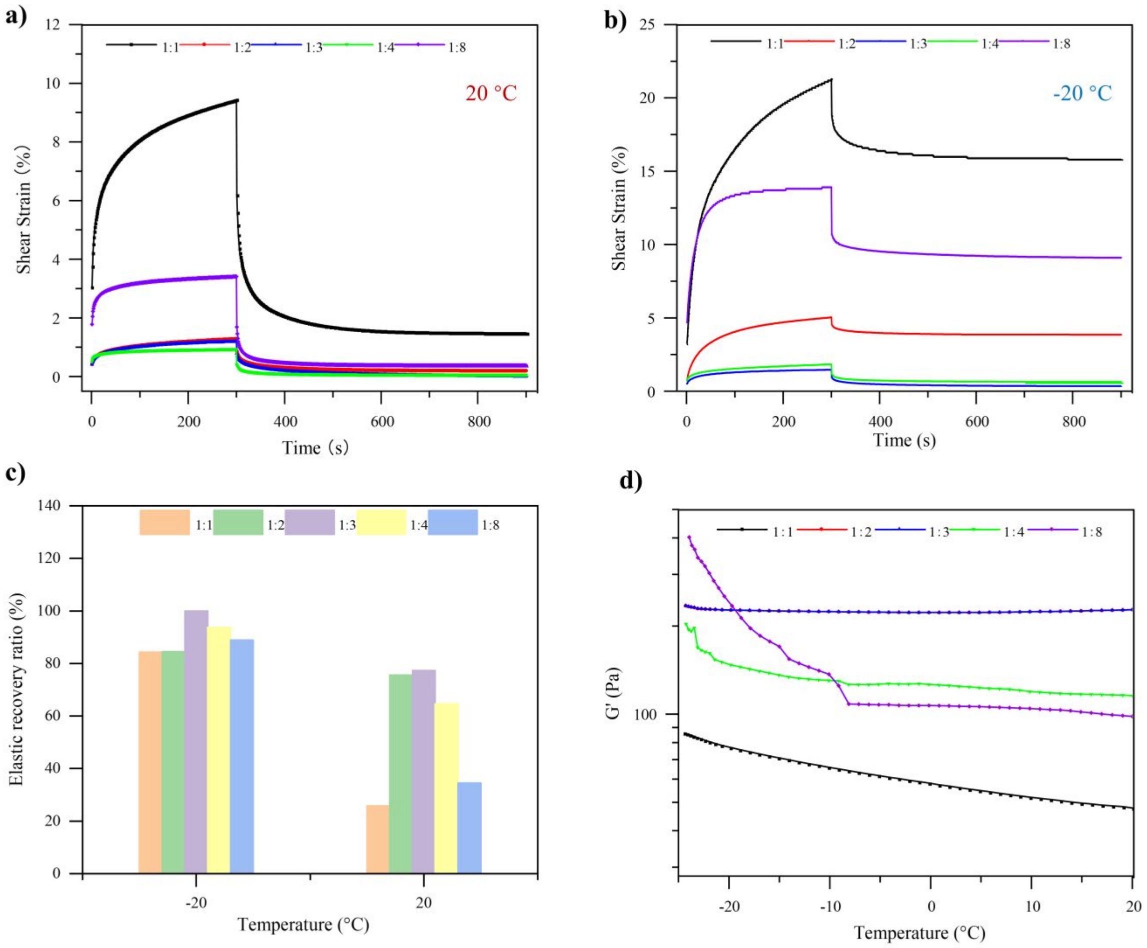

Figure S5. (a-b) The creep recovery tests of the hydrogels with solvent of different ratios of glycerol and PSS ionic liquid in this work. A shear stress of 5 Pa was applied to each hydrogel for 5 min followed by 10 min of recovery at 20 °C and -20 °C. (c) Comparison of elastic recovery behavior of hydrogels with varying glycerol-to-ionic liquid ratios at 20 °C and -20 °C. (d) Storage modulus of hydrogels from 20 °C to -30 °C.

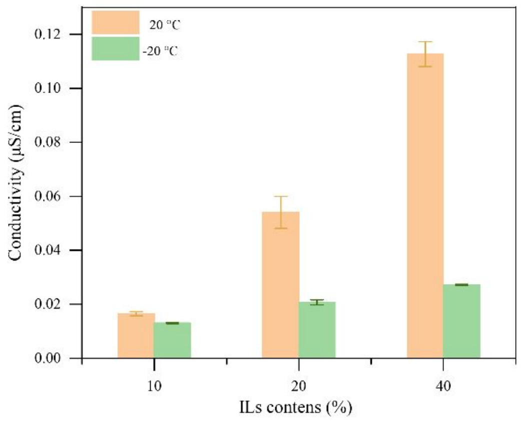

Figure S6. Conductivity of the DGPSS-glycerol at 20 °C and -20 °C.

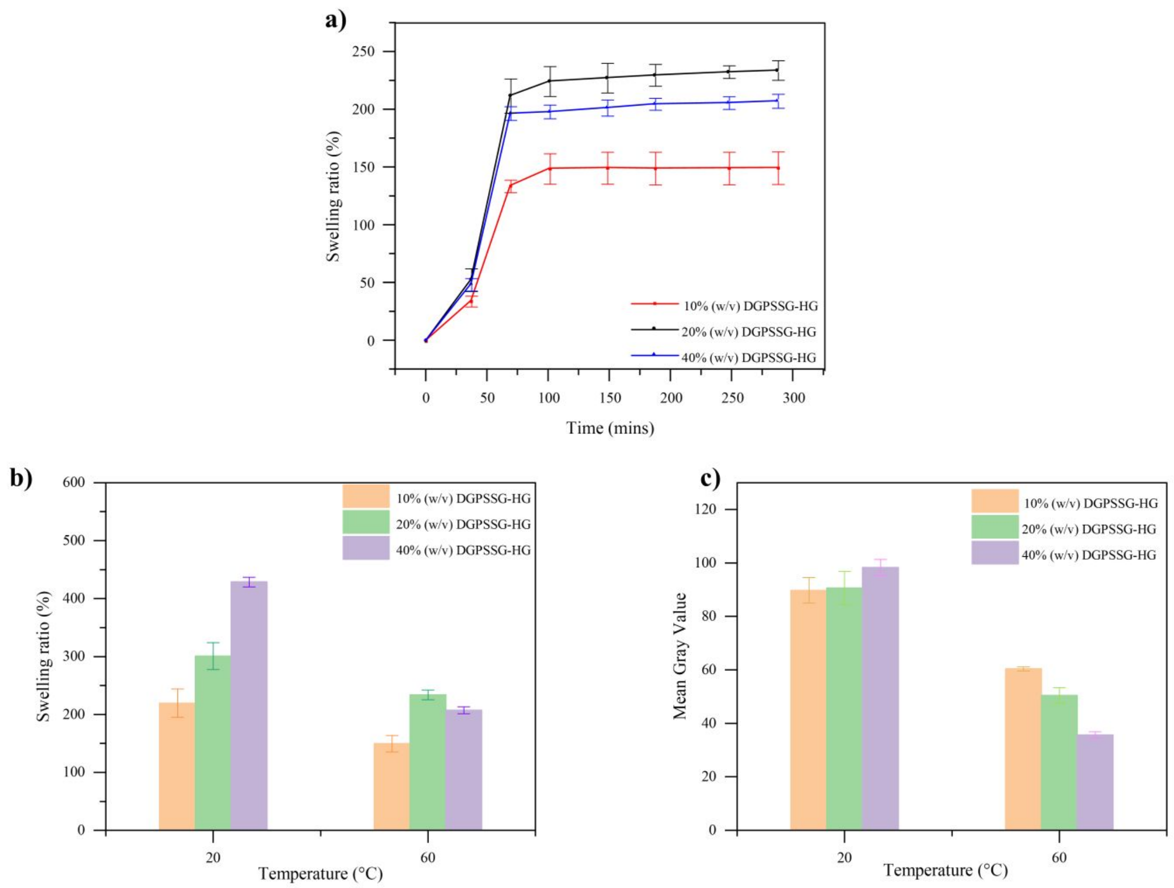

Figure S7. (a) Swelling ratios of DGPSSG hydrogels with different ionic liquid contents at 60 °C.(b) The comparison of swelling ratios of the DGPSSG at 20 °C and 60 °C. (c) Mean gray value of DGPSSG hydrogels with different ionic liquid contents at 20 °C and 60 °C.

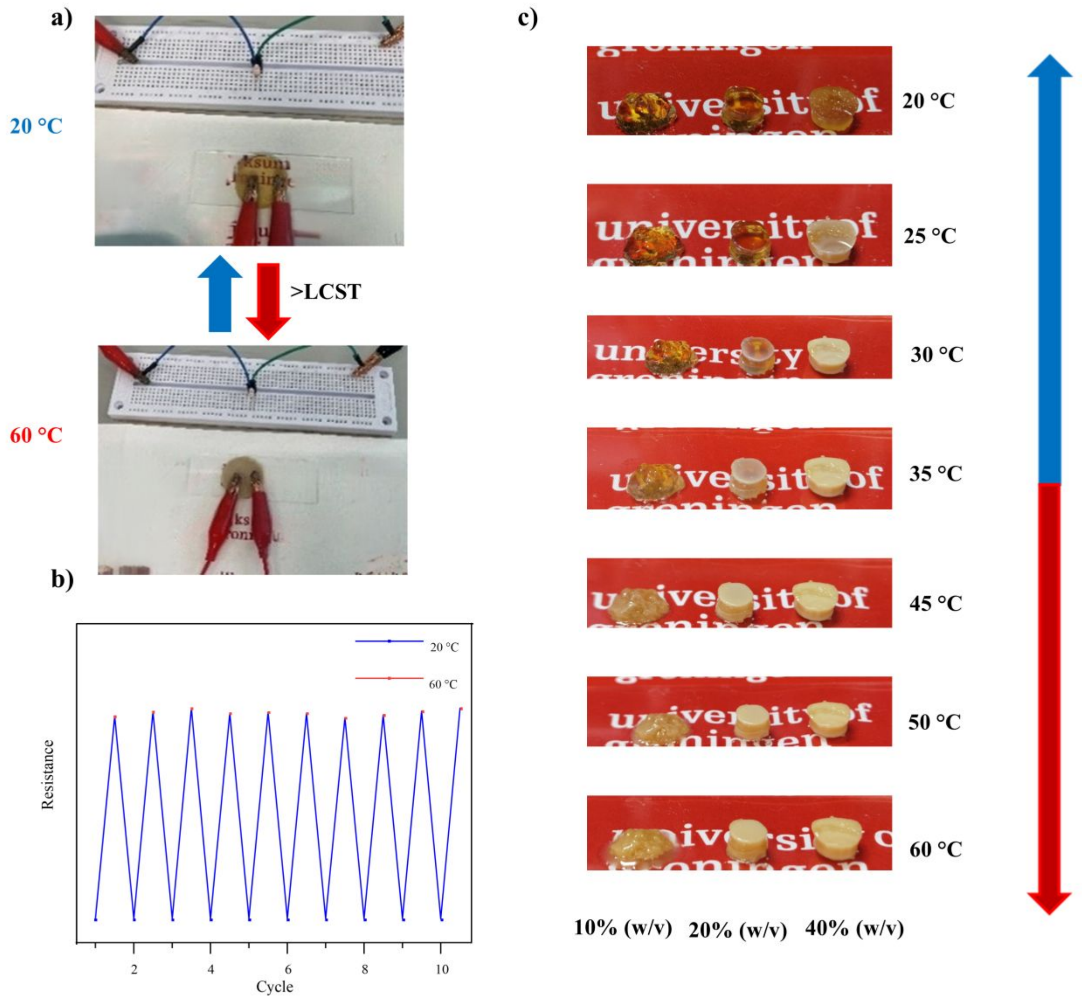

Figure S8. (a) Photographs of the setup for the conductivity measurements of DGPSS-glycerol hydrogel and DGPSS hydrogel at 20 °C and 60 °C; (b) resistance variations during repetitive cold (20 °C) and heat (60 °C) source approach. (c) photographs of the DGPSSG hydrogel's cylinders at different temperatures (from 20 to 60 °C).

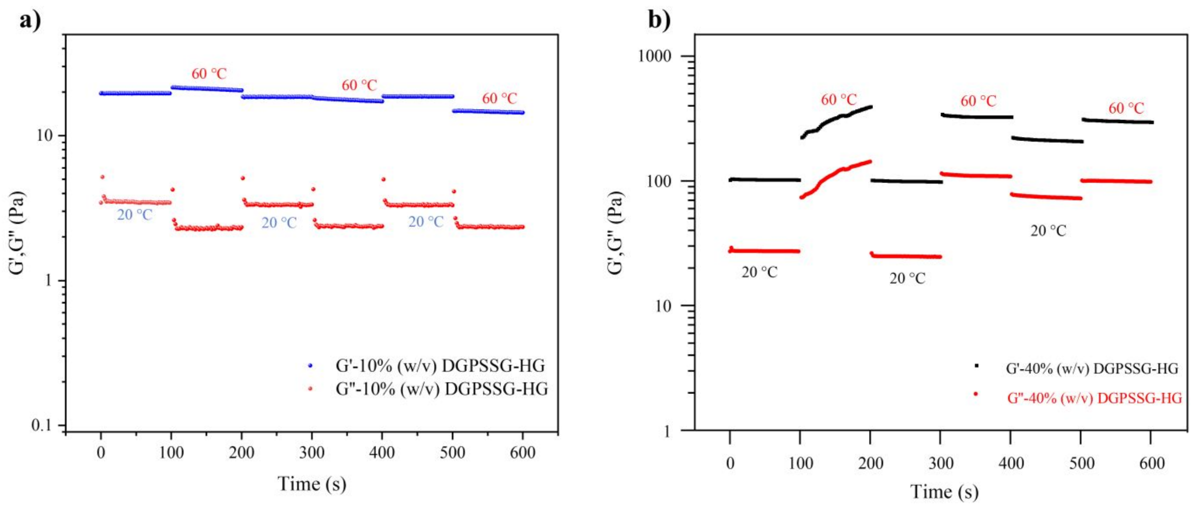

Figure S9. (a-b) Dynamic stepwise temperature amplitude tests of DGPSSG hydrogel at different temperatures.

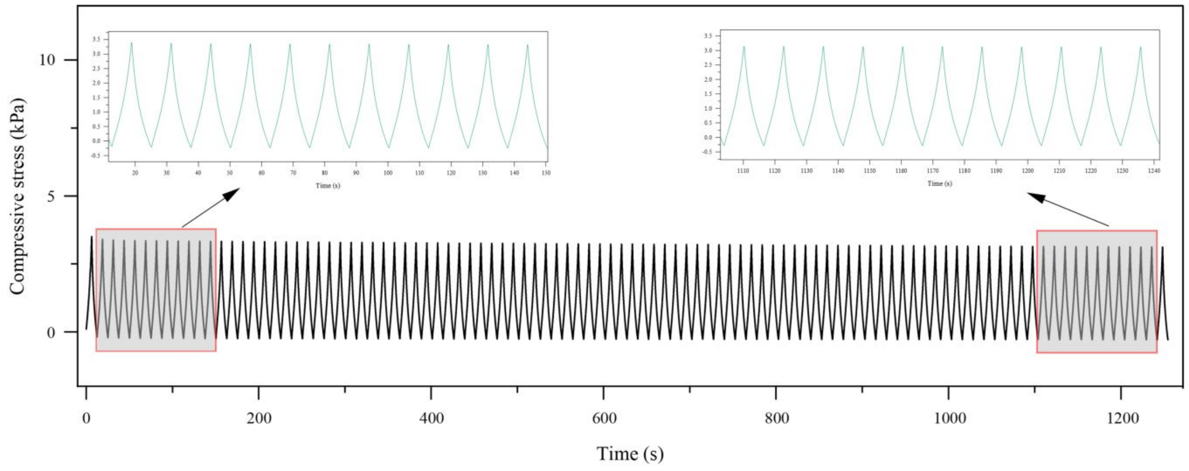

Figure S10. Compressive stress-strain curves of the hydrogel under 100 consecutive cycles at 30% strain at -20 °C.

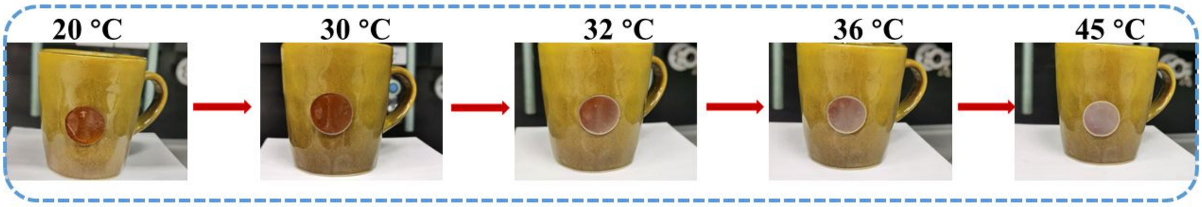

Figure S11. DGPSSG hydrogels for temperature sensors in a certain range (from 20 °C to 45 °C).
